# Temporal Changes in the Function of Bacterial Assemblages Associated With Decomposing Earthworms

**DOI:** 10.3389/fmicb.2021.682224

**Published:** 2021-08-11

**Authors:** Yao-Qin Sun, Yuan Ge

**Affiliations:** ^1^State Key Laboratory of Urban and Regional Ecology, Research Center for Eco-Environmental Sciences, Chinese Academy of Sciences, Beijing, China; ^2^University of Chinese Academy of Sciences, Beijing, China

**Keywords:** bacterial community, decomposition, dissolved organic matter, earthworm, structure–function relationship

## Abstract

Soil invertebrate corpse decomposition is an ecologically significant, yet poorly understood, process affecting nutrient biogeochemical cycling in terrestrial ecosystems. Here, we attempted to answer how the substrate chemistry and microbial community change during soil invertebrate (earthworm) decomposition and what roles microbes play in this process. Specifically, the dead earthworms (*Amynthas corticis*) were buried in two soils where the earthworms inhabited, or not, until more than 50% of the earthworm mass was lost. For both soils, earthworms decomposed faster during the early stage (between 0 and 3 days), as reflected by the higher rate of decomposition and increased accumulation of dissolved organic matter (DOM). This decomposition pattern was paralleled by bacterial community dynamics, where bacterial richness and diversity were significantly higher during early decomposition (*p* < 0.05) with the relative abundances of many genera decreasing as decomposition progressed. The succession of the bacterial community composition was significantly correlated with time-course changes in DOM composition (*p* < 0.05). Particularly, more functional groups (e.g., microbes associated with carbon, nitrogen, and sulfur cycling) were identified to be linked with the change of a specific DOM type during the early decomposition phase. By exploring the ecologically important process of soil invertebrate decomposition and its associated bacterial communities, this study provides evidence, e.g., a statistically significant positive correlation between bacterial community and DOM compositions, which supports the widely recognized yet less-tested microbial community structure–function relationship hypothesis in invertebrate decomposition.

## Introduction

Decomposition is a fundamental process to energy flow and nutrient cycling within ecosystems, with the balance between decomposition and net primary production responsible for ecosystem carbon content (Hu et al., 2001). Compared with plant litter, the carbon–nitrogen ratio in dead animals is much lower (animal C:N ≈ 10:1 versus leaf litter C:N ≈ 66:1) (Nardi et al., 2002; Prieto et al., 2019). The higher nutrient concentration and lower chemical recalcitrance in dead animals may promote their microbial degradability, resulting in hot spots of soil nutrient cycling. It is estimated that the decomposition rate is faster for dead animal matter than for plant litter by up to three orders of magnitude (Parmenter and Macmahon, 2009). Also, the hot spots of nutrient cycling resulting from the decomposing animal may have long-term (for years) effects on soil conditions and processes (Strickland and Wickings, 2015; Keenan et al., 2019; Benbow et al., 2020). However, probably due to the relatively low level of biomass compared with plants (0.005 versus 450 gigatons of carbon in terrestrial ecosystems) and the rapid mineralization of animal tissues, studies regarding animal decomposition have historically lagged far behind those focusing on plant litter decomposition (Bar-On et al., 2018; Barton et al., 2019; Herzog et al., 2019; Benbow et al., 2020). The dynamic process, especially the role of microbes in the process, of dead animal decomposition is poorly understood.

Earthworms, composed of approximately 398 genera and 5,358 species^1^, account for up to 90% of invertebrate biomass present in soil (Edwards, 2004). As a soil-dwelling animal, earthworms spend their whole life cycle within the soil. Over the past 600 million years (Pasha et al., 2018), the coevolution of earthworms with the various environments has resulted in their robust adaptation and survival. The estimated density of earthworms is 5 to 150 individuals per square meter across the globe (Phillips et al., 2019). When an earthworm dies, the immune system ceases to function (Metcalf et al., 2016), and the indigenous microbes (in both gut and body surface) may have the advantage of gaining immediate access to the readily available nutrients in the host. Additionally, the soil microbes may immigrate to this nutrient patch and actively decompose the dead earthworm. For example, microbes can incorporate more than half of the nitrogen from decomposing earthworm tissue into their biomass (Whalen et al., 1999). In a 28-day mesocosm experiment in Malagasy Ferralsol, the decomposing earthworms contributed 30% of plant-available phosphorus increase in soil (Trap et al., 2021). However, it is unclear how the specific bacterial taxa and functional groups change during the decomposition of earthworm tissues.

As earthworm decomposition progresses, substrate quality likely changes due to the activity of successive microbial decomposers. Natural organic matter is derived primarily from plant and animal tissue decay and plays a critical role in the carbon cycle (Nebbioso and Piccolo, 2012). Dissolved organic matter (DOM) refers to the components of natural organic matter that can pass through a 0.45-μm filter pore. These soluble organic substrates are important energy sources for heterotrophic microbial communities. As metabolic preferences and substrate affinities vary among bacteria, changes in bacterial diversity and community composition are likely the prerequisite to optimize utilization of DOM (Gonsior et al., 2016; Li et al., 2018). What is not yet known, however, is how the microbial communities and DOM composition change during the decomposition of animal tissue. Furthermore, the structure–function relationships for the microbial communities are often assumed, but the empirical evidence on this assumption is limited for invertebrate decomposition (Bier et al., 2015; Trivedi et al., 2016; Nannipieri et al., 2017). By exploring soil invertebrate decomposition and the associated bacterial communities, it enables testing of the microbial community structure–function relationship hypothesis, e.g., a statistically significant positive correlation between bacterial community similarity and DOM composition similarity. In addition to testing the hypothesis, this study can also advance the understanding of the earthworm decomposition process and identify the key players.

In this study, we investigated the dynamic process by which microbes self-assemble on decaying earthworms, and we asked: (1) how the substrate chemistry and bacterial communities change during earthworm decomposition and (2) what roles bacteria play in the decomposition process. We hypothesized that the total concentration of DOM would first increase and then decrease, but with different magnitudes of change for various components, thus causing the changes in DOM composition during earthworm decomposition. Prior to these shifts in DOM, the bacterial community composition may also change. For example, nitrogen-cycling-related functional groups, stimulated by the protein-rich decomposing earthworm, would dominate in the initial stage of decomposition and then would be replaced by generalists (e.g., carbon-cycling related group) as decomposition proceeds. Thus, the microbial community structure–function relationship would be observed during the decomposition of earthworm tissues.

## Materials and Methods

### Earthworm Decomposition Experiment

*Amynthas corticis* is an epi-endogeic earthworm species with global distribution (Cunha et al., 2017). The earthworms were collected using an electroshocking method from grassland in Beijing (39°57′N, 116°17′E). Specifically, four soil probes were placed into the soil in a square plot with a resulting sampling area of 1 m^2^. The probes were part of an electroshocking protocol that was used to stimulate earthworms emerging within the sampling area (Weyers et al., 2008). Electroshocking has been shown to have minimal effects on soil biota (Staddon et al., 2003). Species identification of the earthworm individuals was based on morphological characteristics such as body color, first dorsal pore, clitellum position, and the number of body segments (Zhao et al., 2015). For those individuals that were identified as *A. corticis*, only intact and mature adult earthworms with similar body size (about 3-g fresh weight) were retained for use in the experimental setup in order to circumvent the effects of minor factors (e.g., integrity and age) on results. Target individuals were brought to the laboratory, and non-targets were returned to the field. Then the target earthworms were surface-washed with sterile water and euthanatized by freezing at −40°C for 10 min (Zhang et al., 2010).

To clarify whether the functioning microbes in decomposition were primarily from the indigenous microbes of earthworm or microbes dwelling in the soil, two soils were collected from geographically distant sites: Beijing (39°57′N, 116°17′E), where the earthworms were originally collected from, and Xuchang (34°02′N, 113°81′E), which is a non-native environment for the collected earthworms. The soils collected from Beijing and Xuchang were denoted as native soil and non-native soil, respectively. Both soils were classified as fluvo-aquic (Zou et al., 2015; Zhao et al., 2019) but with different bacterial community compositions due to the geographical separation (Supplementary Table 1 and Supplementary Figure 1). Theoretically, if the microbes taking part in the decomposition are derived from soil, this will result in different decomposition dynamics and decomposer communities for the native soil and non-native soil treatments; however, if indigenous earthworm microbes are the primary decomposers, then similar decomposition dynamics and decomposer communities will be observed. At each sampling site, the soil was collected from the top 20 cm of the field in five 1 m × 1 m plots. In each plot, 20 soil cores (5-cm diameter) were randomly collected. All collected soil samples were thoroughly homogenized and immediately transported to the laboratory on ice. The samples were then stored at 4°C for less than 2 weeks before use.

For each microcosm, sieved (mesh size 2 mm) and homogenized soil (120-g dry weight equivalent) was placed in polypropylene bottles (12.5-cm height, 6-cm diameter). Three euthanatized earthworms were put in a nylon mesh bag (mesh size < 1 mm), and their weights were recorded. The mesh bags allowed for efficient sampling and nutrient exchange. Then the decomposition bag containing dead earthworms was buried in the middle layer of soil (4 cm from the bottom), mimicking a realistic situation. Soil moisture was adjusted to 40% of water-holding capacity by adding sterile deionized water. All microcosms were incubated at 20°C in the dark in a climate chamber. Based on a preliminary experiment, approximately 80% of earthworm mass is lost in native soil due to decomposition within 10 days. Therefore, to ensure sufficient samples for subsequent analysis, the incubation period was set as 8 days. To explore earthworm decomposition dynamics and bacterial community shifts, five replicates were destructively sampled on days 0, 1, 3, 5, and 8 for the following DNA extraction and DOM analyses. In total, 50 decomposition bags (2 soil types × 5 time points × 5 replicates = 50) and 50 soil samples (collected underneath the decomposing earthworm) were collected.

### Earthworm Mass Loss Measurement

The mass loss was determined from the difference of dry weight between the initial and final mass of each sampling time. At each sampling time, decomposition bags were removed from the microcosms and were freeze-dried at −40°C for 24 h to a constant weight using a vacuum freeze dryer (Labconco, Kansas City, MO, United States). After drying, the undigested soil in earthworm gut was carefully removed from earthworm tissues using tweezers. Then the tissue samples were weighed and manually ground to fine powder with a mortar. The freeze-dried samples were also used for subsequent DOM analysis and DNA extraction. Because fresh earthworms were used for the decomposition experiment, the initial tissue dry mass (three euthanatized earthworms) in each microcosm was estimated based on the ratio of tissue dry mass to total wet mass (including tissue dry mass, dry mass of undigested soil in gut, and water). To determine the ratio, three replicates of fresh earthworms collected from the same batch were freeze-dried, and the tissue dry mass of these three earthworms was determined following the same procedure described above to calculate the proportion of tissue dry mass to total wet mass.

### Earthworm Dissolved Organic Matter Analysis

Freeze-dried earthworm powder (0.02 g) was dissolved in ultrapure water with a ratio of 1:10. The suspensions were centrifuged at 8,000 *g* and 4°C for 10 min and then filtered through a 0.45-μm cellulose acetate membrane. The collected filtrate was diluted 20-fold with ultrapure water to an appropriate concentration (fluorescence intensity < 10,000) before excitation-emission matrix (EEM) spectroscopy analysis. The EEM analysis is a widely used tool to characterize the composition of complex DOM mixture (Chen et al., 2003), since different DOM components emit different fluorescence intensities with specific emission wavelengths when excited by specific excitation wavelengths. EEM fluorescence spectra were measured on an F-4500 fluorescence spectrophotometer (Hitachi, Tokyo, Japan) with a xenon excitation source. EEM scans were made at the excitation range from 200 to 400 nm and an emission range from 280 to 500 nm at 5-nm increments. The scanning rate was set at 12,000 nm min^–1^. The spectrum of ultrapure water was recorded as the blank (Zhang et al., 2016). Raman scattering, which can result in distortion of the measured spectrum due to energy change, was removed by subtracting the fluorescence spectra of the blank. EEM fluorescence spectra were classified into five fractions to represent five major types of DOM (Chen et al., 2003): tyrosine-like proteins (region 1; excitation, 200–250 nm; emission, 280–330 nm), tryptophan-like proteins (regions 2; excitation, 200–250 nm; emission, 330–380 nm), fulvic acid-like organics (region 3; excitation, 200–250 nm; emission, 380–550 nm), microbial byproduct-like materials (region 4; excitation, 250–400 nm; emission, 280–380 nm), and humic acid-like organics (region 5; excitation, 250–400 nm; emission, 380–550 nm).

### Assessing Microbial Community

Total DNA was extracted from 0.2 g of earthworm powder samples and soil samples from each of the replicates, for each treatment and sampling time, using the QIAGEN DNeasy PowerSoil Kit (QIAGEN, Hilden, Germany). The quality of DNA was assessed using the ND-1000 spectrophotometer (NanoDrop, Wilmington, DE, United States). Primer pairs 338F (5′-ACT CCT ACG GGA GGC AGC AG-3′) and 806R (5′-GGA CTA CHV GGG TWT CTA AT-3′), targeting the V3–V4 region of 16S RNA genes, were used for amplification (Tian et al., 2018). An additional 6-base barcode was added to 806R (Sampson et al., 2016). The barcode was unique to each sample and permitted the identification of individual samples within a single MiSeq sequencing run. We performed three PCR technical replicates per sample on each DNA extract. The 20-μl PCRs were conducted using 0.5 μl of each primer (10 mM), 10 μl of Premix Taq (1.25 U of DNA polymerase, 0.4 mM of dNTPs, 3 mM of Mg^2+^; Takara, Dalian, China), 1 μl of template DNA (10 ng), and 8 μl of nuclease-free PCR-grade water. The PCR was performed on a T100 Thermal Cycler (Bio-Rad, Hercules, CA, United States). The thermal cycling conditions were as follows: 94°C for 5 min, followed by 28 cycles of 94°C for 30 s, 55°C for 30 s, 72°C for 45 s, and a final extension at 72°C for 10 min. Negative controls, other ingredients not changed except the DNA templates replaced with sterilized water, were included for detecting potential contamination (Sun et al., 2019). PCR products were pooled and purified with the AxyPrep DNA Gel Extraction Kit (Axygen, Hangzhou, China). The barcode-tagged amplicons were mixed in equimolar concentrations for MiSeq library construction and then subjected to 250 base pair (bp) paired-end sequencing on the MiSeq platform (Illumina, San Diego, CA, United States) in a commercial laboratory (Majorbio, Beijing, China).

The resulting sequence reads were first sorted based on orientation, and the barcodes were extracted by using extract.barcodes.py in QIIME (Caporaso et al., 2010). Then the sequences were demultiplexed using the q2-demux plugin in QIIME 1.91 (Caporaso et al., 2010). PCR primers were trimmed using Cutadapt allowing for one mismatch (Martin, 2011). The trimmed reads were merged using the FLASH 2 (minimum overlap, 10; max overlap, 65; max mismatch density, 0.25), and quality filtering was performed with the fastq_filter command (Magoc and Salzberg, 2011). Paired reads were discarded if the average number of base error in the read (expected errors) was greater than 2. PCR chimeras were filtered out using USEARCH (Edgar, 2010). Then, identical sequences were dereplicated, and the singletons with possible erroneous sequences were removed using USEARCH (Edgar, 2010). The remaining high-quality sequences were clustered into operational taxonomic units (OTUs) using UCLUST at a 97% sequence similarity cutoff (Shi et al., 2019). The OTUs were assigned taxonomy in QIIME using the SILVA ribosomal RNA gene database project (Quast et al., 2012). All non-bacterial sequences were removed.

### Statistical Analyses

We estimated the temporal dynamics of mass loss and the bacterial alpha diversity of decomposing earthworms using linear regression analysis. The slopes of linear fits were compared by bootstrapping followed by *t*-test (Efron and Tibshirani, 1986; Zhou et al., 2008). The differences for mass loss, bacterial richness, Simpson index, and the amount of DOM at specific sampling time in native soil and non-native soil were compared using independent-samples *t*-test. To understand how the DOM composition changed during decomposition, principal coordinate analysis (PCoA) was performed based on the Bray–Curtis distance of the DOM fluorescence spectra. Also, permutational multivariate analysis of variance (PERMANOVA) was conducted to test the significant distinctions in the DOM constituent across different sampling times.

Principal coordinate analysis based on the Bray–Curtis matrix was performed to explore changes in the bacterial community composition, and the shift patterns were confirmed by PERMANOVA. To examine how the identity of bacterial taxa changed during earthworm decomposition, the shared genera between different sampling times and the unique genera at a specific sampling time were visualized via a network graph using Gephi v0.9.2 (Bastian et al., 2009). To further examine how the abundance of specific bacterial genera changed during earthworm decomposition, we used linear regression to test for the significant trends over time. Fast expectation-maximization microbial source tracking (FEAST), a source tracking method, was used to quantify the contribution of the likely source environments (source) to the microbial composition on decaying earthworm (Shenhav et al., 2019). Mantel test, based on Pearson’s correlations between the two distance matrices (e.g., Bray–Curtis distance of DOM composition), was performed to test whether the decomposition process depends on shifts in the microbial community (Ge et al., 2016).

Potential functions for bacterial taxa were evaluated with the program “functional annotation of prokaryotic taxa” (FAPROTAX). The FAPROTAX defines the metabolic functions in terms of prokaryotic taxa (e.g., genera or species) (Louca et al., 2016). Currently, 90 functional groups covering the process of carbon, nitrogen, and hydrogen cycling (e.g., methylotrophy, nitrate respiration, and hydrocarbon degradation) are included in the database. To identify the specific functional group that regulated the decomposition process, a correlation matrix was constructed by calculating Pearson’s correlation coefficients between the abundance changes for a bacterial functional group and the changes in DOM fluorescence intensity. The changes of bacterial functional group abundance and DOM were calculated by subtracting their values at day 0 from the values at each sampling time. When the abundance of functional group changed in concert with the changes in DOM amount, positive correlations would be detected. In contrast, negative correlations would be observed when the abundance of functional group and the amount of DOM changed in opposite ways. The Benjamini–Hochberg method was used to adjust the original *p*-values of correlation analysis to control the false discovery rate caused by multiple testing. PCoA, PERMANOVA, and Mantel test were conducted using the “vegan” package in R platform^2^.

## Results

### Decomposition Rate Decreases Over Time

**FIGURE 1:**
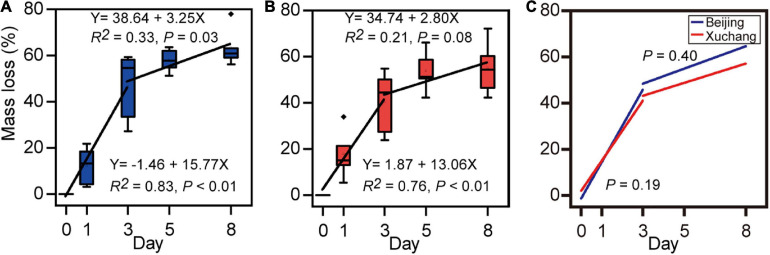
Loss of earthworm mass in native soil **(A)** and non-native soil **(B)**, and the comparison of decomposition rates between native soil (Beijing) and non-native soil (Xuchang) **(C)**. Decomposition rates were estimated as the slope of a linear regression of mass loss versus time.

After 8 days of incubation, a decrease in the total mass of approximately 60% was detected (Figure 1A, 63% in native soil; Figure 1B, 55% in non-native soil). There were no significant differences in mass loss between native soil and non-native soil (*p* = 0.33). At each sampling time, the mass loss was also unaffected by soil origin (Supplementary Table 2). Decomposition rates, which were estimated as the slope of a linear regression of mass loss versus time, showed similar temporal trends in native soil (Figure 1A) and non-native soil (Figure 1B). In native soil, the decomposition rate before day 3 was 15.77 and then decreased to 3.25 (Figure 1A). In non-native soil, the decomposition rate before day 3 was 13.06 and then decreased to 2.80 (Figure 1B). A significant increase in decomposition rate was detected before day 3 (*p* < 0.01 for both soils); then the decomposition rate significantly decreased after day 3 (Figure 1A, *p* = 0.03; Figure 1B, *p* = 0.08). However, there was no significant difference in decomposition rate for soil either before day 3 (*p* = 0.19) or after day 3 (*p* = 0.40).

### Dissolved Organic Matter Composition Shifts Along the Process of Decomposition

**FIGURE 2:**
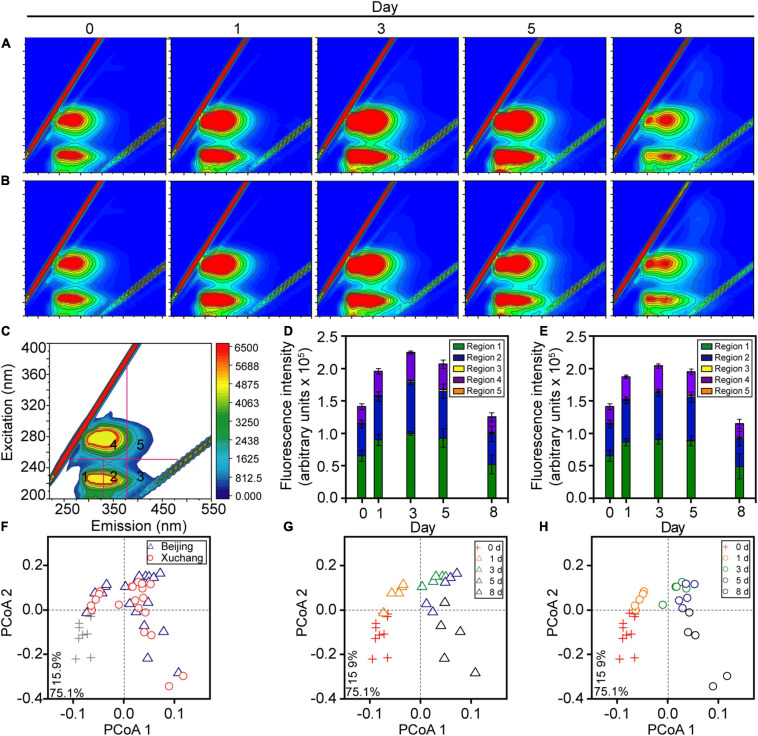
The dissolved organic matter (DOM) composition of decaying earthworm changed during decomposition in native soil and non-native soil. Representative excitation-emission spectra of DOM in native soil **(A)** and non-native soil **(B)**. Five fractions in excitation-emission spectra **(C)**: tyrosine-like proteins (region 1), tryptophan-like proteins (region 2), fulvic acid-like organics (region 3), microbial byproduct-like materials (region 4), and humic acid-like organics (region 5). The color refers to increasing of fluorescence intensity from blue to red in **A–C**. The amount of total DOM and all five fractions showed a clear temporal pattern either in native soil **(D)** or non-native soil **(E)**. The shifts of DOM composition over time were visualized in the principal coordinate analysis (PCoA; **F–H**) based on the Bray–Curtis distance of excitation-emission spectra. The triangles indicate the samples in native soil (Beijing), circles indicate those in non-native soil (Xuchang), and the crosses indicate the samples in native soil and non-native soil at day 0.

Throughout the study period, the total amount of earthworm DOM showed a clear temporal pattern in both native and non-native soils, with a distinct increase before day 3 (*p* < 0.01) and a subsequent decrease after day 3 (Figures 2A,B, *p* < 0.01). More specifically, in native soil, the fluorescent intensities of all five DOM types increased during the first 3 days (*p* < 0.01), with a peak of ∼2.2 × 10^5^ arbitrary units on day 3 and then a decrease to ∼1.3 × 10^5^ arbitrary units by day 8 (Figure 2D, *p* < 0.01). In non-native soil, the fluorescent intensities increased to a peak of ∼2.0 × 10^5^ arbitrary units during the first 3 days (*p* < 0.01) and then decreased to ∼1.1 × 10^5^ arbitrary units over time (Figure 2E, *p* < 0.01). Both in native soil and non-native soil, the extracted DOM was dominated by tyrosine-like proteins (region 1, about 45%), tryptophan-like proteins (region 2, about 35%), and microbial byproduct-like materials (region 4, about 18%, Figure 2 and Supplementary Table 3). The amount of total DOM and specific DOM types was not affected by soil origin for most sampling times, except day 3 (Supplementary Table 4, *p* > 0.05 for all except the tyrosine-like proteins, tryptophan-like proteins, and microbial byproduct-like materials at day 3).

The shifts in DOM composition over time were also visualized in the PCoA based on the Bray–Curtis distance, and the significance of differences was confirmed by PERMANOVA. The decomposition time had a significant effect on DOM composition in both soils (*p* < 0.01), as shown by the progressive changes of DOM composition over time in native soil (Figure 2G) and non-native soil (Figure 2H). Similar to the results of DOM amount, DOM composition was not affected by soil origin for most sampling times, except day 3 (Figure 2F and Supplementary Table 5, *p* > 0.05).

### Bacterial Diversity Changes in Different Decomposition Phases

**FIGURE 3:**
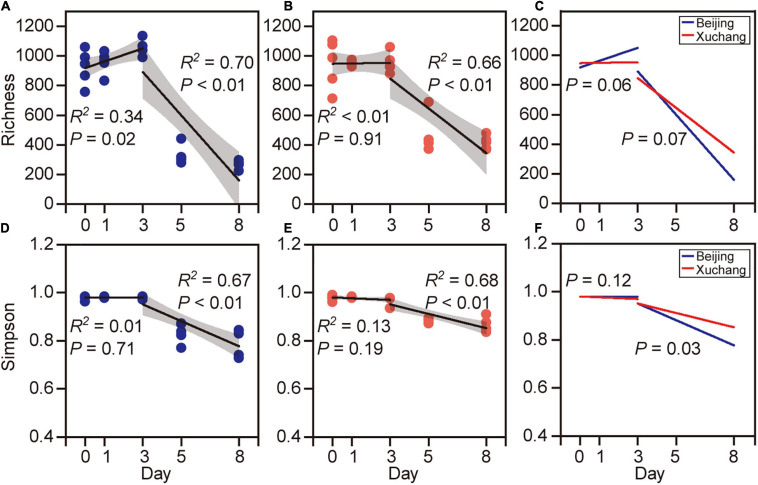
The richness [number of observed operational taxonomic units (OTUs)] and Simpson index of bacterial communities on decaying earthworm. Bacterial diversity showed clear temporal pattern in both native soil treatment **(A,C)** and non-native soil treatment **(B,D)**. The richness **(A,B)** and Simpson index **(C,D)** of bacterial communities showed higher values before day 3 and then decreased after day 3. The temporal changes of bacterial richness were marginally different between native soil and non-native soil either before day 3 (**E**, *p* = 0.06) or after day 3 (**E**, *p* = 0.07). The Simpson index was significantly different in two soils after day 3 (**F**, *p* = 0.03).

Bacterial community richness and Simpson index showed higher values before day 3 and then decreased thereafter (Figure 3). In native soil, the richness increased initially during the first 3 days (*p* = 0.02) and then decreased (*p* < 0.01, Figure 3A). In non-native soil, the richness did not change initially (*p* = 0.91) and then decreased during later stages of decomposition (*p* < 0.01, Figure 3B). The Simpson index is another characteristic of diversity, with a value range between 0 and 1. A value closer to zero indicates that one or a few species dominate, while a value closer to 1 indicates that the number of each species in the community is relatively equal and that more species are present in the sample. The Simpson index showed no significant change with time before day 3 in either native (*p* = 0.71, Figure 3C) or non-native (*p* = 0.19, Figure 3D) soils, whereas this index declined significantly after day 3 (*p* < 0.01 for both soils). The temporal changes of bacterial richness were marginally different in both soils either before day 3 (*p* = 0.06) or after day 3 (*p* = 0.07, Figure 3E). The Simpson index displayed a significant difference between the soils only after day 3 (*p* = 0.03, Figure 3F). For each sampling time, the bacterial richness was significantly affected by soil origin except on day 1 (Supplementary Table 6, *p* < 0.05 for all except day 1). The Simpson index was significantly affected by soil origin only on days 5 and 8 (Supplementary Table 6).

**FIGURE 4:**
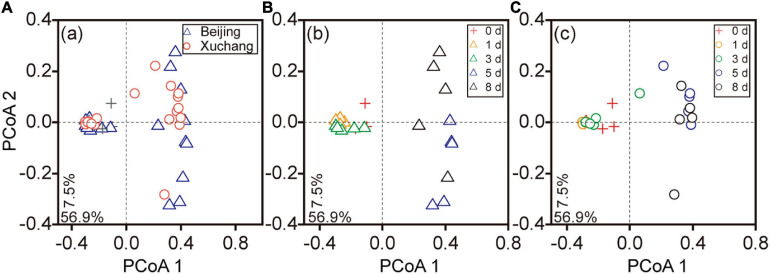
The principal coordinate analyses (PCoAs) based on the Bray–Curtis distance of bacterial communities at different sampling times. Triangles indicate the samples in native soil (Beijing), circles indicate those in non-native soil (Xuchang), and the crosses indicate the samples in native soil and non-native soil at day 0. PCoA of bacterial communities in native and non-native soil treatments **(A)**. Decomposition time had a significant effect on bacterial community composition in native soil (**B**, *p* < 0.01) as well as in non-native soil (**C**, *p* < 0.01).

The succession of the bacterial community on the decaying earthworms was also clearly detected, as indicated by PCoA performed on the entire dataset (Figures 4A–C). The communities of the first 3 days were clustered together and well separated from the bacterial communities of days 5 and 8 in both native soil and non-native soil (Figures 4B,C). Decomposition time had a significant effect on bacterial community composition in the native soil treatment (Figure 4B, *p* < 0.01) as well as in the non-native soil treatment (Figure 4C, *p* < 0.01). The bacterial communities displayed significant differences between native soil and non-native soil on most sampling days (Supplementary Table 7, *p* < 0.05 for all except day 8).

### Bacterial Taxa Changes in Different Decomposition Phases

**FIGURE 5:**
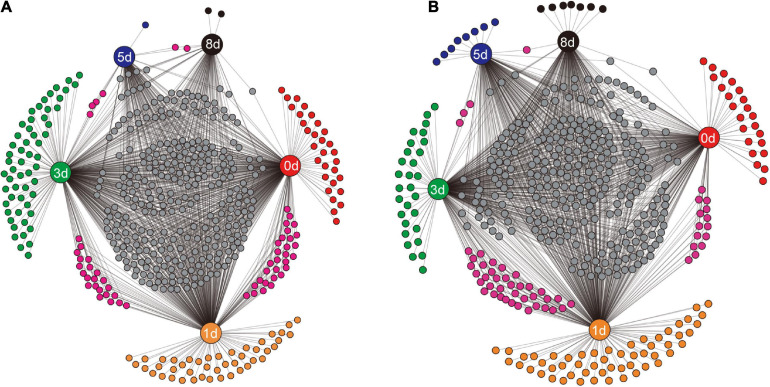
The shared genera between different sampling times and the unique genera at a specific sampling time in native soil treatment **(A)** and non-native soil treatment **(B)**. The sampling times (days 0, 1, 3, 5, and 8) are indicated by the nodes with labels. Each unlabeled node represented an individual genus. Connections (black lines) were drawn between specific sampling time and the related genera.

To examine how the identity of bacterial taxa changed during decomposition, the shared genera between different sampling times, and the unique genera at specific sampling times, were visualized (Figure 5). In the native soil (Figure 5A), the decaying earthworms supported more unique genera during the first 3 days, 30, 44, and 56 genera. While fewer unique genera, one (*Proteobacterium*) and two (*Oscillochloris* and *Terrisporobacter*), were present on days 5 and 8, respectively. The unique genera in the non-native soil (Figure 5B) showed a similar pattern to that in native soil with the number of unique genera before day 3 being 23, 54, and 29 genera, and then dropping to eight (e.g., *Brevibacillus* and *Romboutsia*) and seven (e.g., *Streptosporangium* and *Allocatelliglobosispora*) on days 5 and 8, respectively. As decomposition proceeded, the number of genera shared by the two adjacent days also decreased. The number dropped to four and two, from 29 and 22 genera in native soil (Figure 5A), and decreased to three and one, from 14 and 37 genera in non-native soil (Figure 5B).

**FIGURE 6:**
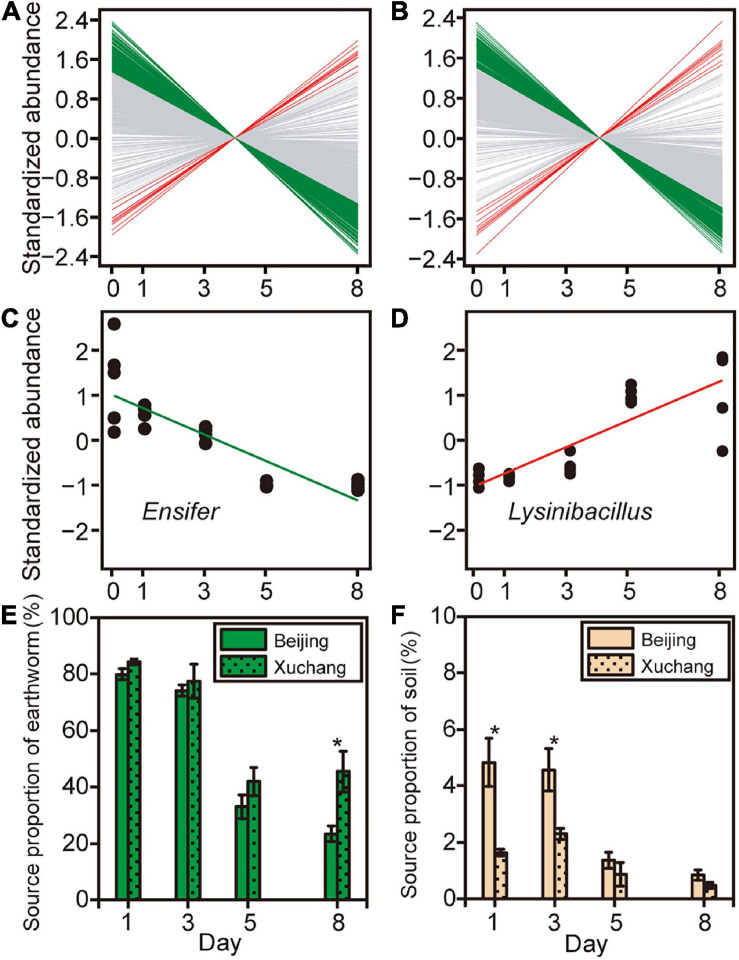
The abundance of specific bacterial genera changed as decomposition proceeded in native soil **(A)** and non-native soil **(B)**. Green lines indicate that the abundance of bacterial genus significantly decreased with time (*p* < 0.05), red lines indicate that the abundance of bacterial genus that significantly increased (*p* < 0.05), and gray lines indicate that there was no significant change in bacterial abundance over time (*p* > 0.05). Two representative genera, *Ensifer* **(C)** and *Lysinibacillus* **(D)**, were selected to show the relationships between taxonomic abundance and decomposition time. Source tracking was used to quantify the proportion of the likely source environments (source), earthworm-derived **(E)** and soil-derived **(F)**, to the microbial community on decaying earthworm. Asterisks indicate significant differences in the contribution of likely source between native soil and non-native soil treatments (independent samples *t*-test, *p* < 0.05).

To further examine how the abundance of specific bacterial genera changed during earthworm decomposition, we used linear regression to test for significant trends over time. Overall, 28% of 744 genera in the native soil treatment and 22% of 729 genera in the non-native soil treatment exhibited significant differential abundance as decomposition proceeded (Figures 6A,B and Supplementary Table 8). Specifically, the relative abundances of more genera on decaying earthworm decreased as decomposition proceeded, 194 in native soil and 149 in non-native soil. In contrast, only a few genera increased in abundance during decomposition, 11 in native soil and nine in non-native soil (representative genera shown in Figures 6C,D). These results suggested the abundance of more taxa on decaying earthworms decreased with decomposition time.

Source tracking was used to quantify the contribution of the likely source environments (source) to the microbial community on decaying earthworms (Figures 6E,F). The “soil” referred to the soil-resident microbes derived from incubated soil, which was defined as the soil bacterial community at day 0. The “earthworm” defined the indigenous bacteria in the earthworm, which was denoted as the bacterial community present on earthworm at day 0. Despite some similarities (shared OTUs), there were still considerable differences between soil and earthworm source (Supplementary Figure 3). In the native soil treatment, the earthworm-derived microbes accounted for 79.9% on day 1 and then decreased to 23.4% by day 8 (Figure 6E). In the non-native soil treatment, the earthworm-derived microbes accounted for 84.3% on day 1 and then decreased to 45.4% by day 8 (Figure 6E). The proportion of earthworm-derived microbes in the native soil treatment tended to be lower than in the non-native soil treatment (Figure 6E, *p* < 0.05 on day 8). Among the different compartments analyzed in this study, the contribution of soil-derived microbes was lower than that of earthworm-derived microbes (Figures 6E,F, *p* < 0.05 for all pairs). In the native soil treatment, the soil-derived microbes accounted for 4.8% on day 1 and then decreased to 0.8% (Figure 6F). In the non-native soil treatment, the soil-derived microbes accounted for 1.6% on day 1 and then decreased to 0.5% by day 8 (Figure 6F). In contrast to earthworm-derived microbes, the proportion of soil-derived microbes in the native soil treatment tended to be greater than that in the non-native soil treatment (Figure 6F, *p* < 0.05 on days 1 and 3). This result suggests that earthworm-derived microbes contributed primarily to the decomposition of earthworm tissues.

### Bacterial Community Correlates to the Shifts in Mass Loss and Dissolved Organic Matter Composition

**FIGURE 7:**
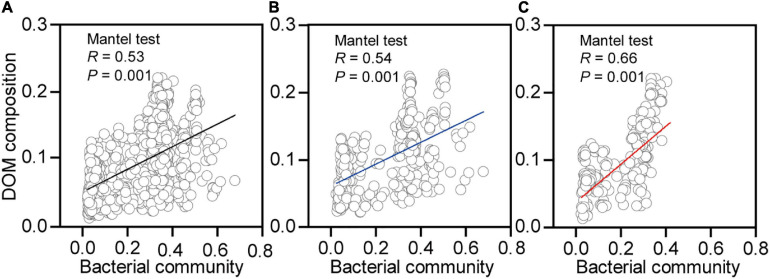
Correlations between bacterial community and dissolved organic matter (DOM) composition for decaying earthworm in both soils **(A)**, native soil treatment (**B**, Beijing), and non-native soil treatment (**C**, Xuchang). The Mantel test (9999 permutations) calculated Pearson’s correlation between the Euclidean distance of bacterial community and Bray–Curtis distance of DOM composition.

Shifts in the DOM composition were significantly correlated to the changes in bacterial community composition for earthworms in native (Figure 7B, *R* = 0.54, *p* = 0.001) and non-native (Figure 7C, *R* = 0.66, *p* = 0.001) soils. The effects of the bacterial community were still maintained when we merged the two datasets from different soils (Figure 7A, *R* = 0.53, *p* = 0.001). In addition, the bacterial community compositions were significantly correlated to mass loss in native (Supplementary Figure 4B, *R* = 0.15, *p* = 0.04) and non-native (Supplementary Figure 4C, *R* = 0.25, *p* = 0.002) soils. The effects of bacterial community on mass loss were still maintained when we merged the two datasets from the different soils (Supplementary Figure 4A, *R* = 0.25, *p* = 0.002).

### Bacterial Functional Groups Associated With Earthworm Decomposition

**FIGURE 8:**
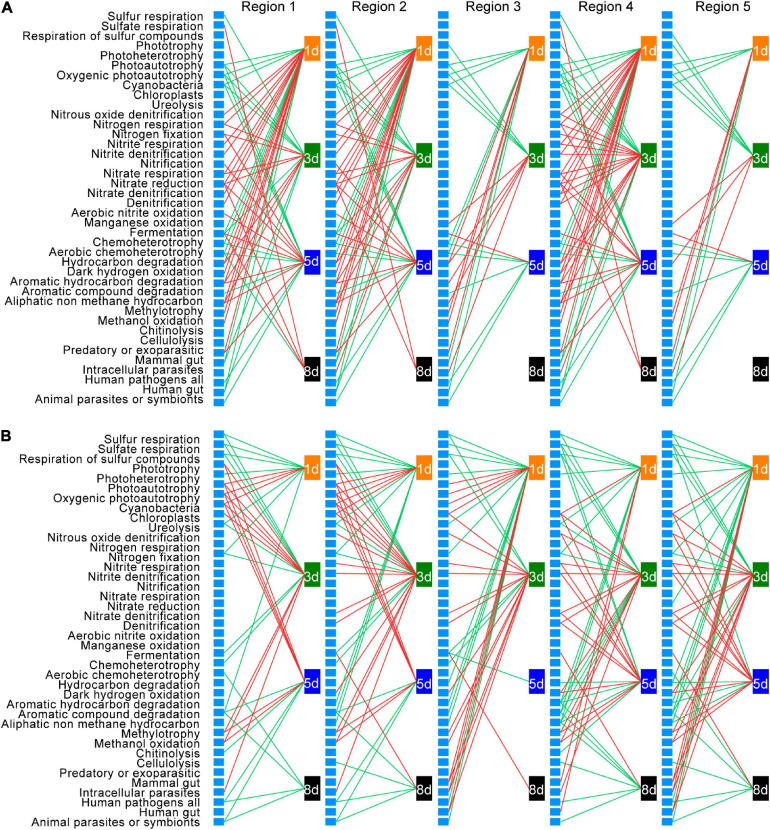
Correlations between changes in the abundance of bacterial functional groups and changes in the amount of dissolved organic matter (DOM) in native soil **(A)** and non-native soil **(B)**. Only significant (*p* < 0.05) correlations are shown. Red lines represent positive correlations, and green lines represent negative correlations. Each unlabeled rectangle represents a specific functional group. Each labeled rectangle represents a specific sampling time. The five groups arranged from left to right are the five types of DOM: tyrosine-like proteins (region 1), tryptophan-like proteins (region 2), fulvic acid-like organics (region 3), microbial byproduct-like materials (region 4), and humic acid-like organics (region 5).

To understand how bacterial functional groups changed during earthworm decomposition, and thus link functional group succession to DOM shift, bacterial taxa were assigned to different functional groups using FAPROTAX database. Then Pearson’s correlation analysis was conducted to explore the relationships between the abundance change of each functional group and the amount change of each DOM type over time (Figure 8 and Supplementary Table 9).

Among the 90 functional groups in the FAPROTAX database, 42 and 40 groups were present in at least one of the samples in the native soil and non-native soil treatments, respectively. After false discovery rate correction, 35 functional groups in native soil and 34 functional groups in non-native soil, whose relative abundance changes were significantly correlated to the amount change of a specific DOM type at a specific sampling time (adjusted *p* < 0.05), were defined as those intimately associated with temporal DOM shift during earthworm decomposition. Among these functional groups, 29 were shared in both native soil and non-native soils. The direction of correlation between the abundance change of specific functional group and the amount change of each DOM type was consistent, within a specific sampling time in each soil treatment. Moreover, the direction of correlations between changes in specific functional group abundance and DOM amount was consistent at different sampling times except for the fermentation group.

We further examined functional group succession during decomposition. Generally, relative to the later time points, a larger number of functional groups were identified to be associated with the change of a specific DOM type in both native soil and non-native soil during the early decomposition phase (Figure 8). This result is consistent with the changes of bacterial richness during earthworm decomposition, further indicating that more bacterial taxa were involved in metabolizing fresh organic matter. Also, based on the phase in which the functional group imposes an effect, the functional groups could be classified into five categories: those associated with DOM changes (1) during early decomposition phase (Figure 8 and Supplementary Figure 5A, 18 and 15 in native and non-native soils, respectively), (2) during middle decomposition phase (Figure 8 and Supplementary Figure 5B, 11 and 10), (3) during late decomposition phase (Figure 8 and Supplementary Figure 5C, one and three), (4) during the entire decomposition process (Figure 8 and Supplementary Figure 5D, two and three), and (5) those irregularly associated with DOM changes during different decomposition phases (Figure 8 and Supplementary Figure 5E, three and three). For example, during the early decomposition phases, the functional groups involved in the nitrogen cycling and methylotrophy were predominant in the native soil treatment, while functional groups related to sulfur cycling, ureolysis, and chemoheterotrophy were predominant in the non-native soil treatment (Supplementary Figure 5A). The functional groups associated with different decomposition phases are shown in Supplementary Figure 5. Particularly, for some shared functional groups in the two soils, there was a time lag in the non-native soil treatment (e.g., methylotrophy and nitrite respiration).

We also examined how the functional groups may mediate the accumulation and consumption of a specific DOM type based on the direction of the correlations. For the 29 functional groups whose abundance changes significantly correlated with the amount change of a specific DOM type in both soils, 16 showed the same direction of correlation in native and non-native soils (nine positive and seven negative), while 13 functional groups showed the opposite directions between the two soils (Figure 8 and Supplementary Figure 5). The positively correlated groups in both soils included those associated with nitrogen cycling, methylotrophy, and dark hydrogen oxidation; and the negatively correlated groups in both soils included those associated with sulfur cycling, chemoheterotrophy, and animal parasites. We also found that six functional groups were significantly and positively correlated with DOM changes in the native soil only, while five functional groups were significantly correlated with DOM changes only in the non-native soil, and most of those correlations (four of five functional groups) were negative.

## Discussion

Animal decomposition, creating resource islands and nutrient pulses, is one of the main drivers of the biogeochemical cycle and consequently has bottom-up importance for ecosystem functions. Studies addressing the role of bacteria during animal decomposition are still rare (Benbow et al., 2019). As far as we know, this is a novel study regarding the microbially mediated process of earthworm decomposition. Given the high biomass of earthworm in soil and highly concentrated nutrients released from decomposing earthworm, it is imperative to explore how the microbial communities mediates the decomposition of earthworm tissues. Further, our study provides support for the hypothesis of microbial community structure–function relationship in invertebrate decomposition. This study will improve our recognition of the importance for the bacterial community in regulating ecosystem function.

We conducted a controlled laboratory study to decipher the microbial-mediated process of earthworm decomposition. After 8 days of incubation in native soil and non-native soil, a decrease in earthworm mass of nearly 60% was detected (Figure 1). The decomposition rate for the earthworm tissues first increased and then decreased in our study (Figures 1A,B), which was similar to the decomposition pattern of the dead vertebrate animal (e.g., pig, rat, and human) (Grassberger and Frank, 2004; Parmenter and Macmahon, 2009; Knobel et al., 2018). The changes in mass loss rate before and after day 3 were likely due to the differences in metabolic preferences and substrate affinities of the decomposers. Previous studies on plant decomposition also showed a similar trend, fast decomposition in the early stage, and then a slow decomposition rate (Horodecki and Jagodzinski, 2019; Wang et al., 2020).

Multiple lines of evidence showed that the bacterial community on decaying earthworm changed as decomposition proceeded. For example, the bacterial richness and Simpson index showed higher values at the early decomposition phase and then decreased (Figure 3). Also, a greater number of genera displayed decreasing relative abundances than those with increasing abundances as decomposition proceeds (Figures 6A,B). These results support one of our hypotheses that bacterial community changes with decomposition time and suggested that more diverse assemblage of microbes participated in metabolizing fresh, protein-rich, organic matter than in metabolic residues. Besides the variation in bacterial identity and abundance, the proportions of likely sources for microbes also changed over time. By using the method of source tracking, we further revealed the microbial sources contributing to the decomposition process (Figures 6E,F). The indigenous microbes (in both gut and body surface) may have the advantage of gaining access to the readily available nutrient in the host during early decomposition; earthworm-derived microbes therefore dominated this stage (Figure 6E). One possible reason why the proportion of earthworm- and soil-source bacteria decreased during the late stage is that the substrate quality was altered by the earlier microbial activity (Gonsior et al., 2016). The changes in substrate composition lead to the decline of some bacteria, freeing up niche space for other opportunistic bacteria to take over (Tobias-Hunefeldt et al., 2020). The source tracking results also explained the differences in the decomposition process in native soil and non-native soil. Since our study focused on the succession of bacterial communities on decaying earthworms and the changes in earthworm substrate composition during decomposition, we did not have treatment without earthworm addition. We used the soil bacterial community at day 0 as the source of the soil-resident bacteria to the microbial community on decaying earthworms, and we found that the soil-derived bacteria contributed a much smaller share than earthworm-derived bacteria (Figures 6E,F, *p* < 0.05). In theory, the bacterial community composition in soil would be different between soils with earthworm additions and those without, because of the nutrients from decomposing earthworm tissues. In this regard, when the study focuses on how soil bacterial communities change during earthworm decomposition, it is best to set controls without earthworm addition.

Although community succession occurred continuously, we identified two discrete phases of decomposition based upon changes in DOM composition and bacterial community diversity over time (Figures 2, 3). The initial stage of decomposition refers to days 0, 1, and 3, while late decomposition stage refers to days 5 and 8. In the present study, the bacteria at the initial stage were clustered together and well separated from the cluster of late-stage samples (Figures 4B,C), indicating a clear temporal pattern of bacterial community succession. The bacterial communities displayed significant differences between native soil and non-native soil on most sampling days (Supplementary Table 7). This result suggested that the bacteria involved in decomposition may also be affected by soil bacteria. The temporal changes of microbial communities in decomposing earthworm were similar to the decomposition pattern of mice (Burcham et al., 2019). Along with these shifts in the bacterial community, the difference in DOM composition progressively increased before day 3, but the differences tended to decrease or not expand after day 3 (Figures 2G,H). These results support our other hypothesis that the composition of DOM changes during earthworm decomposition. Beyond the mass loss, the study extended to the DOM composition of substrates, and we found that the bacterial community was also significantly (Figure 7B, *R* = 0.54, *p* = 0.001; Figure 7C, *R* = 0.66, *p* = 0.001) correlated to the chemical composition of decaying earthworm. Bacterial community composition correlated weakly but significantly with mass loss (Supplementary Figure 4B, *R* = 0.15, *p* = 0.04; Supplementary Figure 4C, *R* = 0.25, *p* = 0.002), while it strongly correlated with DOM composition. These results indicated that microbial communities play an irreplaceable role in the progression of decomposition. These results also provided evidence regarding the microbial structure–function relationship in invertebrate decomposition: the difference in DOM composition increased with the increasing dissimilarity in bacterial community composition.

Microbial taxa vary in their substrate preferences and strategies of nutrient acquisition, whereby some microbes may have higher acquisition ability and thus an advantage in the community (Calderon et al., 2017). Based on functional annotation, using FAPROTAX, and correlation analysis between changes of the each functional group abundance and the amount for different DOM types, we found that in both native soil (Figure 8A) and non-native soil (Figure 8B), a greater number of bacterial taxa, belonging to different functional groups, drove the decomposition process during the early phase. These results support our third hypothesis that the microbial community structure correlates with the decomposition dynamics of earthworm tissues. Among the functional groups associated with DOM changes during early decomposition phase, the groups such as the nitrate respiration and methylotrophy were detected in native soil, while groups such as ureolysis and chemoheterotrophy appeared in non-native soil (Supplementary Figure 5A). Functional groups such as aromatic compound degradation and methylotrophy were detectable during middle decomposition phase in native soil and non-native soil, respectively (Supplementary Figure 5B). The late decomposition phase was characterized by functional groups involved in sulfate respiration in native soil, chemoheterotrophy, fermentation, and cellulolysis in non-native soil (Figure 8 and Supplementary Figure 5C). As for which functional groups performed differently in the two soils (positive versus negative, early phase versus late phase), it may depend on the metabolic process and the state of the metabolites. When the metabolic process is complete, the final metabolites may escape into the air, resulting in a negative correlation. When the process is still ongoing, the products exist as metabolic intermediates, and positive correlations may be detected. Furthermore, the soil microbes may also modulate the metabolic process.

The main limitation of applying FAPROTAX to our data is the implicit assumption in FAPROTAX that if the cultured members of a taxon can perform a particular function, then all members of the taxon (both the cultured and non-cultured) can perform that function (Ge et al., 2018). However, less than 1% of all microbial taxa have been studied in culture. In this sense, the functional implications of the shifts in bacterial community could be larger than what can be currently inferred using this approach (Ge et al., 2018). With the 16S sequencing approach, we used function-by-proxy to determine the components of the structure–function relationship; however, our ability to parse functional groups is limited. A functional group can only be assigned when the bacteria taxa satisfy the following requirements: (1) can be matched with a known nearest relative in a database; and (2) the known taxa can be matched with a function in FAPROTAX. While we focused on the role of bacteria, the roles of other biotic factors (e.g., fungi, protozoa, mites, and nematodes) were not taken into account in the decomposition process. In reality, it is important to note that soil fauna also plays a key role in the decomposition. In addition, the abiotic factors (e.g., temperature, light, and moisture) had been shown to affect the process of carrion and plant litter decomposition (Metcalf et al., 2016; Fabian et al., 2018; Maenhout et al., 2018; Zhang et al., 2019). In our study, to clarify the role of bacteria in the decomposition process, abiotic factors (e.g., temperature and light) were kept constant, and their effects on earthworm decomposition were not taken into consideration.

## Conclusion

As far as we know, this is a novel study regarding the microbial-mediated process of earthworm decomposition by applying high-throughput sequencing. In this controlled laboratory study, we found that earthworms decomposed faster during the early stage (between 0 and 3 days), as reflected by the higher rate of decomposition and increased accumulation of DOM. We also found that the succession of the bacterial community composition was significantly correlated with time-course changes in DOM composition. In fact, the decomposition of soil animal tissue is a complex process because of many interacting factors related to the chemical, physical, and biological properties of the earthworm, as well as to soil environment and climate. This study provides evidence supporting the microbial community structure–function relationship hypothesis in invertebrate decomposition.

## Data Availability Statement

The datasets presented in this study can be found in online repositories. The names of the repository/repositories and accession number(s) can be found below: https://www.ncbi.nlm.nih.gov/, PRJNA637816.

## Author Contributions

YG designed research. Y-QS performed the research. Y-QS and YG analyzed the data and wrote the manuscript. Both authors read and approved the final manuscript.

## Conflict of Interest

The authors declare that the research was conducted in the absence of any commercial or financial relationships that could be construed as a potential conflict of interest.

## Publisher’s Note

All claims expressed in this article are solely those of the authors and do not necessarily represent those of their affiliated organizations, or those of the publisher, the editors and the reviewers. Any product that may be evaluated in this article, or claim that may be made by its manufacturer, is not guaranteed or endorsed by the publisher.

## Footnotes

1
http://taxo.drilobase.org/

2
https://www.r-project.org/

## Supplementary Material

Supplementary Figure 1 Principal coordinates analysis plot of Bray-Curtis distance for soil bacterial community in native soil and non-native soil.

Supplementary Figure 2 The fluorescence intensity of earthworm dissolved organic matter (DOM) at different sampling times.

Supplementary Figure 3 The unique and shared OTUs between likely source environments.

Supplementary Figure 4 Correlations between bacterial community and mass loss of decaying earthworm in both soils, native soil treatment, and non-native soil treatment.

Supplementary Figure 5 The specific bacterial functional groups associated with decomposition phase in native and non-native soils.

Supplementary Table 1 The physico-chemical properties of native soil and non-native soil.

Supplementary Table 2 Significance test of mass loss between native soil and non-native soil at specific sampling times.

Supplementary Table 3 The proportion of different dissolved organic matter (DOM) types at specific sampling times in native soil treatment and non-native soil treatment.

Supplementary Table 4 Significance test for the amount of different dissolved organic matter (DOM) types between native soil and non-native soil at specific sampling times.

Supplementary Table 5 Significance test of dissolved organic matter (DOM) composition between native soil and non-native soil at specific sampling times.

Supplementary Table 6 Significance test of bacterial community richness and Simpson index on decaying earthworm between native soil and non-native soil treatments at specific sampling times.

Supplementary Table 7 Significance test of bacterial community composition on decaying earthworm between native soil treatment and non-native soil treatment at specific sampling times.

Supplementary Table 8 List of specific genera for which the abundance significantly changed during earthworm decomposition in native soil treatment and non-native soil treatment.

Supplementary Table 9 The specific bacterial functional groups associated with earthworm decomposition at different sampling times.
