# Supplementary material for: Temporal Changes in the Function of Bacterial Assemblages Associated With Decomposing Earthworms
Source: Front Microbiol. 2021 Aug 11;12:682224. doi: 10.3389/fmicb.2021.682224 (PMC8386022; doi:10.3389/fmicb.2021.682224)
Supplement: Supplementary Figure 1 — Principal coordinates analysis plot of Bray-Curtis distance for soil bacterial community in native soil and non-native soil. [file Data_Sheet_1.docx]

Temporal changes in the function of bacterial assemblages associated with decomposing earthworms

Yao-Qin Sun ^1,2^, Yuan Ge ^1,2,*^

1. State Key Laboratory of Urban and Regional Ecology, Research Center for Eco-Environmental Sciences, Chinese Academy of Sciences, Beijing 100085, China

2. University of Chinese Academy of Sciences, Beijing 100049, China

Corresponding author

Yuan Ge, Research Centre for Eco-Environmental Sciences, Chinese Academy of Sciences, 18 Shuangqing Road, Beijing 100085, China. Tel: (86) 10 62913536, E-mail: yuange@rcees.ac.cn.

Number of pages: 32

Number of figures: 5

Number of tables: 9


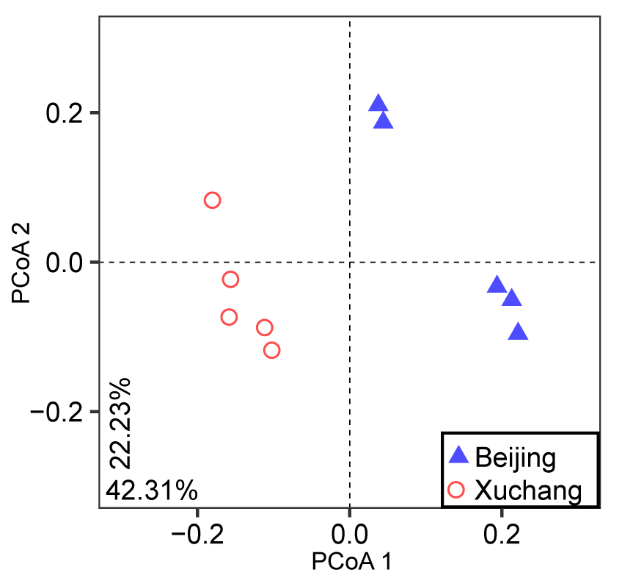


**Fig. S1** Principal coordinates analysis plot of Bray-Curtis distance for soil bacterial community in native soil (Beijing) and non-native soil (Xuchang). The significance of difference was analyzed by permutational multivariate analysis of variance (PERMANOVA) with 999 permutations. There was a significant difference in the bacterial community between native soil and non-native soil (*P* < 0.01).


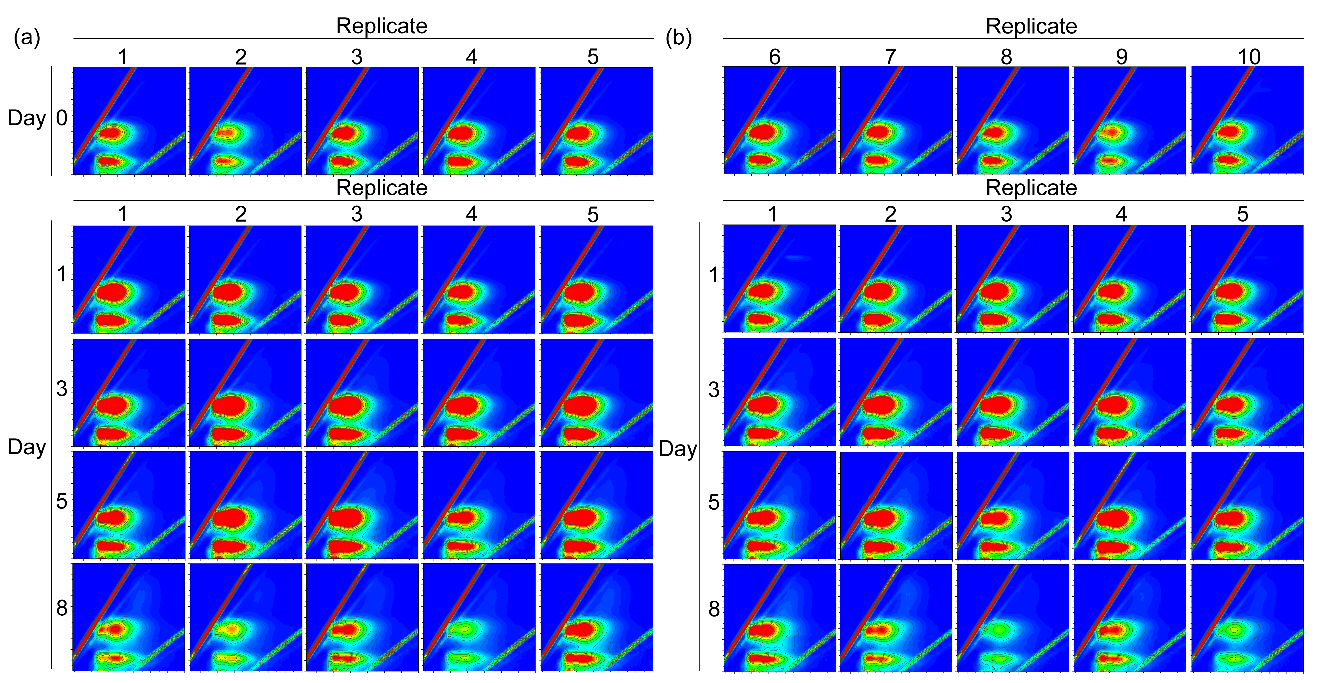


**Fig. S2** The fluorescence intensity of earthworm dissolved organic matter (DOM) at different sampling times. There were 10 replicates at day 0 and 5 replicates, each, on days 1, 3, 5, and 8. There was no necessity to distinction between two soil treatments for the earthworm samples at day 0, due to the dead earthworms were not buried in soils at day 0. The color refers to increasing of fluorescence intensity from blue to red. The fluorescence intensity of DOM showed a clear temporal pattern either in native soil (a) or non-native soil (b), with a distinct increase before day 3 and a subsequent decrease after day 3 (*P* < 0.01).


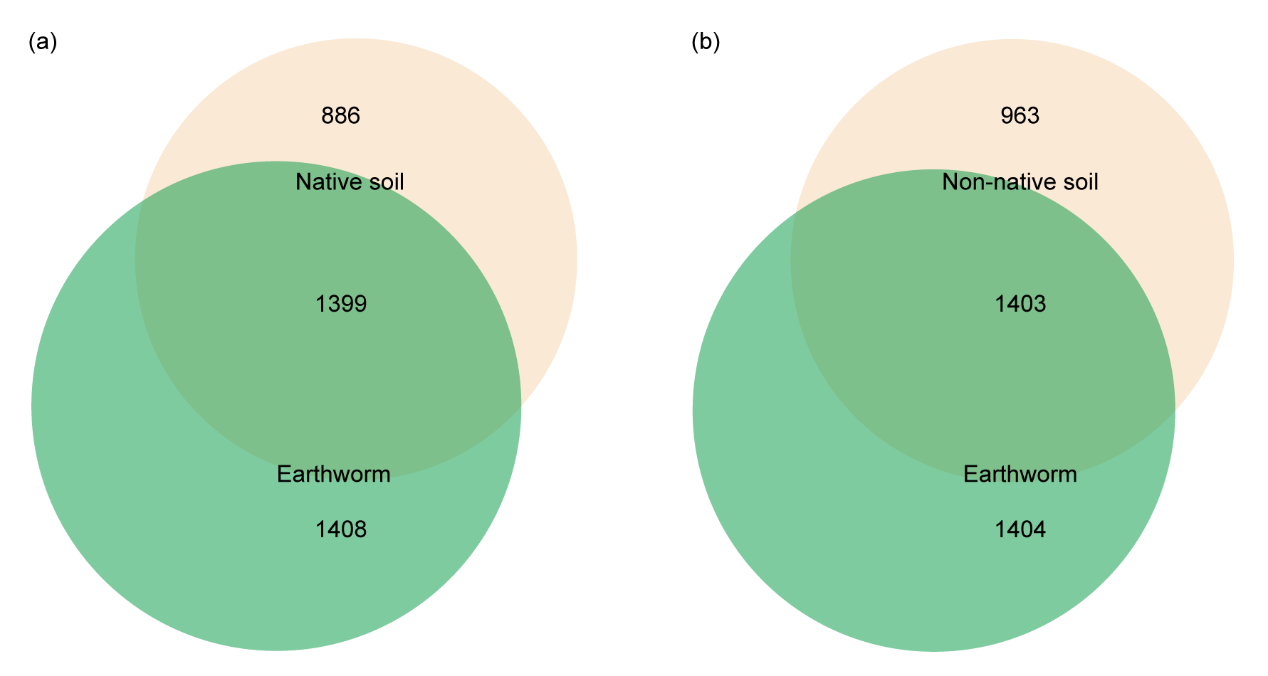


**Fig. S3** The unique and shared OTUs between likely source environments. Venn diagram showing the unique and shared OTUs between soil and earthworm source. For the native soil treatment (a), 1408 (38.13%) OTUs were unique to the earthworm, 886 (23.99 %) OTUs were unique to the earthworm, and shared OTUs were 1399 (37.88%) between native soil and earthworm. For the non-native soil treatment (b), 1404 (37.24%) OTUs were unique to the earthworm, 963 (25.54%) OTUs were unique to the earthworm, and shared OTUs were 1403 (37.21%) between native soil and earthworm.


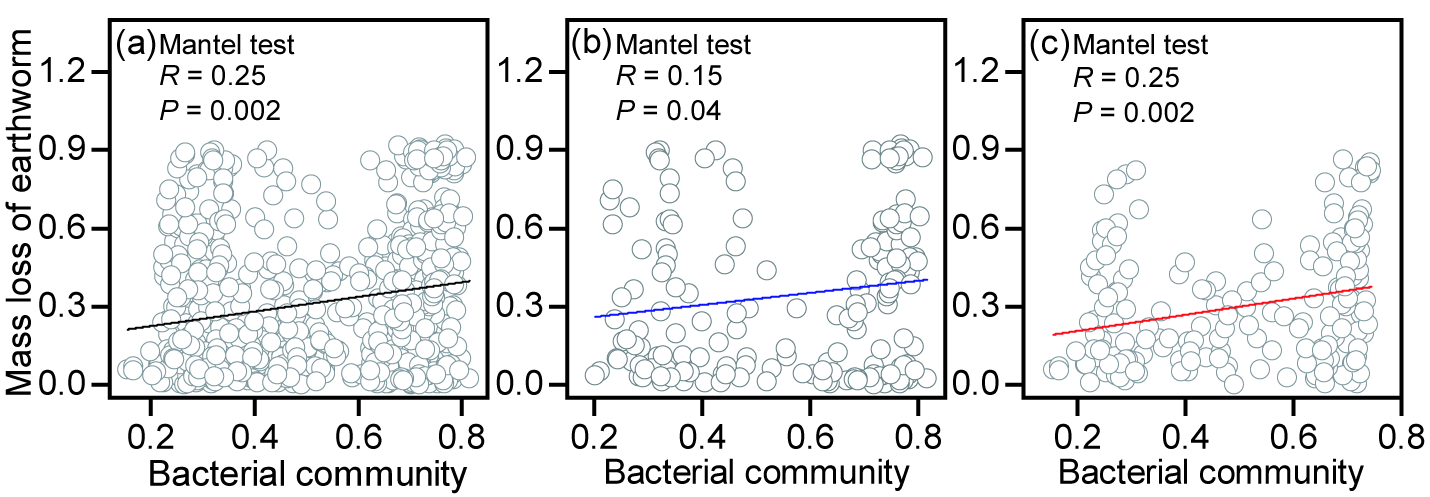


**Fig. S4** Correlations between bacterial community and mass loss of decaying earthworm in both soils (a), native soil treatment (b, Beijing) and non-native soil treatment (c, Xuchang). The Mantel test (9999 permutations) calculated the Pearson correlation between the Bray-Curtis distance of bacterial community and the mass loss.


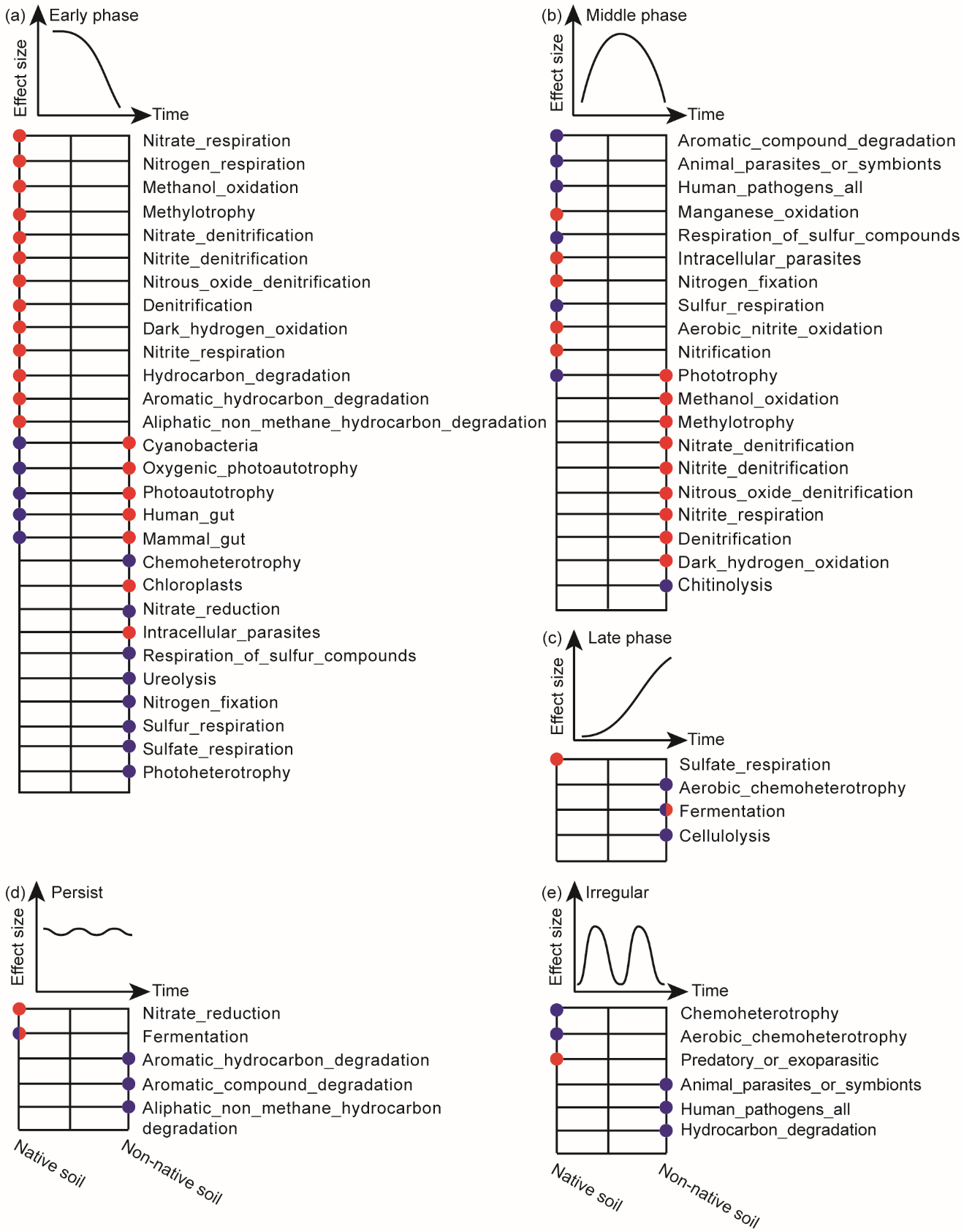
**Fig. S5** The specific bacterial functional groups associated with different decomposition phases in native and non-native soils. Red dots indicate positive correlations between the abundance changes of bacterial functional group and amount changes of dissolved organic matter (DOM), blue dots indicate negative correlations. Dot with half red and half blue indicates the direction of correlation changed from negative to positive. Based on the phase in which the bacterial functional group imposes an effect, the functional groups could be classified into different categories: 1) those associated with DOM changes at early decomposition phase (a), 2) at middle decomposition phase (b), 3) at late decomposition phase (c), 4) during the entire decomposition (d), and 5) irregularly associated with DOM changes during the decomposition (e). Generally, more functional groups were identified to be associated with the change of a specific DOM type in both native soil and non-native soil at the early decomposition phase.

**Table S1** The physico-chemical properties of native soil and non-native soil.

| Property | Native soil | Non-native soil |
| --- | --- | --- |
| Soil type | fluvo-aquic soil | fluvo-aquic soil |
| pH | 7.62 ± 0.03 | 7.55 ± 0.07 |
| Total carbon (g kg^-1^) | 23.02 ± 2.61 | 17.59 ± 0.37 |
| Total nitrogen (g kg^-1^) | 1.32 ± 0.13 | 1.34 ± 0.01 |
| NH_4_^+^-N (mg kg^-1^) | 5.11 ± 2.17 | 8.48 ± 3.82 |
| NO_3_^-^-N (mg kg^-1^) | 4.70 ± 0.99 | 8.90 ± 0.15 |

Values represent mean ± standard deviation, n = 5.

**Table S2** Significance test of mass loss between native soil and non-native soil at specific sampling times (days 1, 3, 5, and 8) using independent samples *t*-test.

| Mass loss | Native vs. Non-native | | | |
| --- | --- | --- | --- | --- |
|  | Day 1 | Day 3 | Day 5 | Day 8 |
| *F* | 0.14 | 0.24 | 1.52 | 0.68 |
| *P* | 0.38 | 0.50 | 0.40 | 0.24 |

**Table S3** The proportion of different dissolved organic matter (DOM) types at specific sampling times (days 1, 3, 5, and 8) in native soil treatment and non-native soil treatment.

| DOM composition (%) | Native soil | | | | | Non-native soil | | | | |
| --- | --- | --- | --- | --- | --- | --- | --- | --- | --- | --- |
|  | Reg 1 | Reg 2 | Reg 3 | Reg 4 | Reg 5 | Reg 1 | Reg 2 | Reg 3 | Reg 4 | Reg 5 |
| Day 1 | 46.10  44.25  45.43  41.84 | 34.47  35.26  34.53  38.11 | 0.71  1.03  1.23  1.59 | 18.55  19.22  18.51  18.04 | 0.17  0.25  0.29  0.43 | 45.85  44.46  44.92  41.58 | 34.12  34.80  34.88  37.78 | 0.73  0.99  1.19  1.54 | 19.13  19.50  18.72  18.70 | 0.18  0.25  0.28  0.42 |
| Day 3 |  |  |  |  |  |  |  |  |  |  |
| Day 5 |  |  |  |  |  |  |  |  |  |  |
| Day 8 |  |  |  |  |  |  |  |  |  |  |

Reg 1: region 1; tyrosine-like proteins, Reg 2: region 2; tryptophan-like proteins, Reg 3: region 3; fulvic acid-like organics, Reg 4: region 4; microbial byproduct-like materials, Reg 5: region 5; humic acid-like organics.

**Table S4** Significance test for the amount of different dissolved organic matter (DOM) types between native soil and non-native soil at specific sampling times (days 1, 3, 5, and 8) using independent samples *t*-test. Significant differences are indicated by bold numbers.

| DOM composition | Native vs. Non-native | | | |
| --- | --- | --- | --- | --- |
|  | Day 1 | Day 3 | Day 5 | Day 8 |
| Total amount (five regions) | 0.45 | **0.03** | 0.52 | 0.66 |
| Region 1 | 0.48 | **0.02** | 0.58 | 0.77 |
| Region 2 | 0.58 | **0.06** | 0.47 | 0.57 |
| Region 3 | 0.36 | 0.33 | 0.81 | 0.76 |
| Region 4 | 0.26 | **0.04** | 0.48 | 0.57 |
| Region 5 | 0.36 | 0.20 | 0.77 | 0.67 |

Region 1; tyrosine-like proteins, Region 2; tryptophan-like proteins, Region 3; fulvic acid-like organics, Region 4; microbial byproduct-like materials, Region 5; humic acid-like organics.

**Table S5** Significance test of dissolved organic matter (DOM) composition between native soil and non-native soil at specific sampling times (days 1, 3, 5, and 8) using permutational multivariate analysis of variance (PERMANOVA) with 999 permutations. Significant differences are indicated by bold numbers.

| Native vs. Non-native | *F* | *R^2^* | *P* |
| --- | --- | --- | --- |
| Day 1 | 0.68 | 0.08 | 0.48 |
| Day 3 | 5.37 | 0.40 | **0.02** |
| Day 5 | 0.34 | 0.04 | 0.56 |
| Day 8 | 0.29 | 0.03 | 0.59 |

**Table S6** Significance test of bacterial community richness and Simpson index on decaying earthworm between native soil and non-native soil treatments at specific sampling times (days 1, 3, 5, and 8) using independent samples *t*-test. Significant differences are indicated by bold numbers.

| Native vs. Non-native | Richness | Simpson index |
| --- | --- | --- |
| Day 1 | 0.96 | 0.92 |
| Day 3 | **0.03** | 0.22 |
| Day 5 | **0.06** | **0.03** |
| Day 8 | **0.00** | **0.06** |

**Table S7** Significance test of bacterial community composition on decaying earthworm between native soil treatment and non-native soil treatment at specific sampling times (days 1, 3, 5, and 8) using permutational multivariate analysis of variance (PERMANOVA) with 999 permutations. Significant differences are indicated by bold numbers.

| Native vs. Non-native | *F* | *R^2^* | *P* |
| --- | --- | --- | --- |
| Day 1 | 2.54 | 0.24 | **0.01** |
| Day 3 | 2.24 | 0.22 | **0.007** |
| Day 5 | 5.85 | 0.42 | **0.007** |
| Day 8 | 2.04 | 0.20 | 0.09 |

**Table S8** List of specific genera for which the abundance significantly changed during earthworm decomposition in native soil treatment and non-native soil treatment.

|  | Soil | Decrease or Increase | Taxonomy |
| --- | --- | --- | --- |
| Genus 2 | Native | Decrease | *Sphingomonadaceae* |
| Genus 3 | Native | Decrease | *Xanthomonadaceae* |
| Genus 4 | Native | Decrease | *Micrococcaceae* |
| Genus 8 | Native | Decrease | *Acidobacteria* |
| Genus 10 | Native | Decrease | *Pyrinomonadaceae* |
| Genus 11 | Native | Decrease | *Burkholderiaceae* |
| Genus 12 | Native | Decrease | *Nitrospiraceae* |
| Genus 14 | Native | Decrease | *Betaproteobacteriales* |
| Genus 15 | Native | Decrease | *Betaproteobacteriales* |
| Genus 18 | Native | Decrease | *Microbacteriaceae* |
| Genus 21 | Native | Decrease | *Acidobacteria* |
| Genus 22 | Native | Decrease | *Rhodanobacteraceae* |
| Genus 23 | Native | Decrease | *Xanthobacteraceae* |
| Genus 24 | Native | Decrease | *Chitinophagaceae* |
| Genus 25 | Native | Decrease | *Burkholderiaceae* |
| Genus 26 | Native | Decrease | *Gemmatimonadaceae* |
| Genus 27 | Native | Decrease | *Gaiellales* |
| Genus 28 | Native | Decrease | *Nocardioidaceae* |
| Genus 31 | Native | Decrease | *Saccharimonadales* |
| Genus 32 | Native | Decrease | *Nitrosomonadaceae* |
| Genus 33 | Native | Decrease | *Acidobacteria Subgroup 6* |
| Genus 36 | Native | Decrease | *Acidobacteria* |
| Genus 38 | Native | Decrease | *Microbacteriaceae* |
| Genus 39 | Native | Decrease | *Sphingomonadaceae* |
| Genus 42 | Native | Decrease | *Acidobacteria Subgroup 6* |
| Genus 45 | Native | Decrease | *Micromonosporaceae* |
| genus46 | Native | Decrease | *Mycobacteriaceae* |
| Genus 49 | Native | Decrease | *Acidobacteria* |
| Genus 52 | Native | Decrease | *Solibacteraceae Subgroup 3* |
| Genus 54 | Native | Decrease | *Acidobacteria Subgroup 17* |
| Genus 56 | Native | Decrease | *Microscillaceae* |
| Genus 57 | Native | Decrease | *Chloroflexi* |
| Genus 62 | Native | Decrease | *Steroidobacteraceae* |
| Genus 64 | Native | Decrease | *Acidobacteria Subgroup 6* |
| Genus 68 | Native | Decrease | *Nitrosomonadaceae* |
| Genus 70 | Native | Decrease | *Betaproteobacteriales* |
| Genus 71 | Native | Decrease | *Myxococcales* |
| Genus 72 | Native | Decrease | *Deltaproteobacteria* |
| Genus 75 | Native | Decrease | *Steroidobacteraceae* |
| Genus 77 | Native | Decrease | *Rhizobiales* |
| Genus 78 | Native | Decrease | *Acidimicrobiia* |
| Genus 80 | Native | Decrease | *Micromonosporaceae* |
| Genus 81 | Native | Decrease | *Rokubacteriales* |
| Genus 82 | Native | Decrease | *Rhizobiaceae* |
| Genus 83 | Native | Decrease | *Actinobacteria* |
| Genus 85 | Native | Decrease | *Microtrichales* |
| Genus 86 | Native | Decrease | *Verrucomicrobiae* |
| Genus 88 | Native | Decrease | *Dongiaceae* |
| Genus 91 | Native | Decrease | *Sandaracinaceae* |
| Genus 93 | Native | Decrease | *Betaproteobacteriales* |
| Genus 94 | Native | Decrease | *Elsterales* |
| Genus 95 | Native | Decrease | *Geodermatophilaceae* |
| Genus 96 | Native | Decrease | *Micrococcales* |
| Genus 101 | Native | Decrease | *Gammaproteobacteria* |
| Genus 102 | Native | Decrease | *Diplorickettsiaceae* |
| Genus 105 | Native | Decrease | *Actinobacteria* |
| Genus 107 | Native | Decrease | *Chloroflexi TK10* |
| Genus 109 | Native | Decrease | *Xanthomonadaceae* |
| Genus 110 | Native | Decrease | *Saprospiraceae* |
| Genus 113 | Native | Decrease | *Deltaproteobacteria* |
| Genus 119 | Native | Decrease | *Chthoniobacteraceae* |
| Genus 125 | Native | Decrease | *Anaerolineae* |
| Genus 130 | Native | Decrease | *Xanthobacteraceae* |
| Genus 131 | Native | Decrease | *Verrucomicrobiaceae* |
| Genus 132 | Native | Decrease | *Rhizobiales* |
| Genus 133 | Native | Decrease | *Solirubrobacterales* |
| Genus 134 | Native | Decrease | *Xanthobacteraceae* |
| Genus 137 | Native | Decrease | *Diplorickettsiaceae* |
| Genus 142 | Native | Decrease | *Gaiellales* |
| Genus 145 | Native | Decrease | *Xanthomonadaceae* |
| Genus 147 | Native | Decrease | *Rhodobacteraceae* |
| Genus 154 | Native | Decrease | *Solirubrobacteraceae* |
| Genus 158 | Native | Decrease | *Acidobacteria Subgroup 17* |
| Genus 163 | Native | Decrease | *Gemmatimonadaceae* |
| Genus 164 | Native | Decrease | *Actinomarinales* |
| Genus 168 | Native | Decrease | *Hevea brasiliensis* |
| Genus 170 | Native | Decrease | *Burkholderiaceae* |
| Genus 171 | Native | Decrease | *Hyphomicrobiaceae* |
| Genus 173 | Native | Decrease | *Chloroflexi* |
| Genus 177 | Native | Decrease | *Planococcaceae* |
| Genus 178 | Native | Decrease | *Geminicoccaceae* |
| Genus 183 | Native | Decrease | *Hyphomonadaceae* |
| Genus 185 | Native | Decrease | *Holophagae* |
| Genus 189 | Native | Decrease | *Rhizobiales Incertae Sedis* |
| Genus 191 | Native | Decrease | *Actinobacteria* |
| Genus 193 | Native | Decrease | *Xanthomonadales* |
| Genus 196 | Native | Decrease | *Bdellovibrionaceae* |
| Genus 197 | Native | Decrease | *Caulobacteraceae* |
| Genus 198 | Native | Decrease | *Sphingobacteriaceae* |
| Genus 201 | Native | Decrease | *Alphaproteobacteria* |
| Genus 203 | Native | Decrease | *Saccharimonadales* |
| Genus 206 | Native | Decrease | *Nocardiaceae* |
| Genus 210 | Native | Decrease | *Rhizobiaceae* |
| Genus 211 | Native | Decrease | *Tepidisphaerales* |
| Genus 224 | Native | Decrease | *Nitrosomonadaceae* |
| Genus 226 | Native | Decrease | *Chloroplast* |
| Genus 228 | Native | Decrease | *Saccharimonadaceae* |
| Genus 231 | Native | Decrease | *Acidobacteria Subgroup 9* |
| Genus 233 | Native | Decrease | *Rhodanobacteraceae* |
| Genus 235 | Native | Decrease | *Solirubrobacterales* |
| Genus 244 | Native | Decrease | *Beijerinckiaceae* |
| Genus 246 | Native | Decrease | *Chitinophagaceae* |
| Genus 247 | Native | Decrease | *Solirubrobacterales* |
| Genus 253 | Native | Decrease | *Chitinophagaceae* |
| Genus 255 | Native | Decrease | *Gaiellaceae* |
| Genus 256 | Native | Decrease | *Solibacteraceae Subgroup 3* |
| Genus 277 | Native | Decrease | *Pseudomonadaceae* |
| Genus 279 | Native | Decrease | *Acidimicrobiia* |
| Genus 285 | Native | Decrease | *Iamiaceae* |
| Genus 286 | Native | Decrease | *Burkholderiaceae* |
| Genus 292 | Native | Decrease | *Pseudonocardiaceae* |
| Genus 293 | Native | Decrease | *Xanthobacteraceae* |
| Genus 295 | Native | Decrease | *Chloroflexi* |
| Genus 296 | Native | Decrease | *Chitinophagaceae* |
| Genus 302 | Native | Decrease | *Armatimonadetes* |
| Genus 316 | Native | Decrease | *Babeliales* |
| Genus 317 | Native | Decrease | *Solimonadaceae* |
| Genus 322 | Native | Decrease | *Caulobacteraceae* |
| Genus 323 | Native | Decrease | *Nostocaceae* |
| Genus 327 | Native | Decrease | *Sphingobacteriales* |
| Genus 335 | Native | Decrease | *Chitinophagaceae* |
| Genus 336 | Native | Decrease | *Rhodobacteraceae* |
| Genus 340 | Native | Decrease | *Microbacteriaceae* |
| Genus 341 | Native | Decrease | *Pseudomonadaceae* |
| Genus 352 | Native | Decrease | *Solirubrobacteraceae* |
| Genus 355 | Native | Decrease | *Polyangiaceae* |
| Genus 357 | Native | Decrease | *Vermiphilaceae* |
| Genus 361 | Native | Decrease | *Coxiellaceae* |
| Genus 377 | Native | Decrease | *Rhodobacteraceae* |
| Genus 383 | Native | Decrease | *Parcubacteria* |
| Genus 385 | Native | Decrease | *Solirubrobacterales* |
| Genus 386 | Native | Decrease | *Bacteriovoracaceae* |
| Genus 394 | Native | Decrease | *Patescibacteria* |
| Genus 397 | Native | Decrease | *Frankiales* |
| Genus 406 | Native | Decrease | *Candidatus Ovatusbacter* |
| Genus 436 | Native | Decrease | *Legionellaceae* |
| Genus 439 | Native | Decrease | *Acidobacteria Subgroup 6* |
| Genus 443 | Native | Decrease | *Planococcaceae* |
| Genus 445 | Native | Decrease | *Vermiphilaceae* |
| Genus 449 | Native | Decrease | *Kaistiaceae* |
| Genus 460 | Native | Decrease | *Holophagae* |
| Genus 461 | Native | Decrease | *Enterococcaceae* |
| Genus 471 | Native | Decrease | *Oligoflexaceae* |
| Genus 474 | Native | Decrease | *Bacillales* |
| Genus 481 | Native | Decrease | *Labraceae* |
| Genus 491 | Native | Decrease | *Trueperaceae* |
| Genus 492 | Native | Decrease | *Lactobacillaceae* |
| Genus 494 | Native | Decrease | *Bacillaceae* |
| Genus 499 | Native | Decrease | *Chitinophagaceae* |
| Genus 507 | Native | Decrease | *Solimonadaceae* |
| Genus 508 | Native | Decrease | *Holosporaceae* |
| Genus 525 | Native | Decrease | *Caulobacteraceae* |
| Genus 530 | Native | Decrease | *Acidobacteriales* |
| Genus 536 | Native | Decrease | *Sphingobacteriaceae* |
| Genus 544 | Native | Decrease | *Firmicutes* |
| Genus 548 | Native | Decrease | *Lachnospiraceae* |
| Genus 550 | Native | Decrease | *Sandaracinaceae* |
| Genus 554 | Native | Decrease | *Bifidobacteriaceae* |
| Genus 555 | Native | Decrease | *Acidobacteriaceae Subgroup 1* |
| Genus 565 | Native | Decrease | *Acetobacteraceae* |
| Genus 572 | Native | Decrease | *Acidobacteriia Subgroup 2* |
| Genus 590 | Native | Decrease | *Enterobacteriaceae* |
| Genus 591 | Native | Decrease | *Staphylococcaceae* |
| Genus 619 | Native | Decrease | *Acidobacteriaceae Subgroup 1* |
| Genus 621 | Native | Decrease | *Orbaceae* |
| Genus 634 | Native | Decrease | *Moraxellaceae* |
| Genus 645 | Native | Decrease | *Corynebacteriaceae* |
| Genus 653 | Native | Decrease | *Moraxellaceae* |
| Genus 656 | Native | Decrease | *Enterobacteriaceae* |
| Genus 664 | Native | Decrease | *Mitochondria* |
| Genus 680 | Native | Decrease | *Burkholderiaceae* |
| Genus 688 | Native | Decrease | *Acetobacteraceae* |
| Genus 692 | Native | Decrease | *Spirosomaceae* |
| Genus 704 | Native | Decrease | *Babeliales* |
| Genus 708 | Native | Decrease | *Pseudonocardiaceae* |
| Genus 709 | Native | Decrease | *Bacillaceae* |
| Genus 715 | Native | Decrease | *Moraxellaceae* |
| Genus 716 | Native | Decrease | *Streptococcaceae* |
| Genus 718 | Native | Decrease | *Chloroflexi* |
| Genus 720 | Native | Decrease | *Weeksellaceae* |
| Genus 764 | Native | Decrease | *Solanum melongena* |
| Genus 839 | Native | Decrease | *Micrococcaceae* |
| Genus 856 | Native | Decrease | *Brevibacteriaceae* |
| Genus 858 | Native | Decrease | *Halomonadaceae* |
| Genus 860 | Native | Decrease | *Dysgonomonadaceae* |
| Genus 862 | Native | Decrease | *Holophagaceae* |
| Genus 863 | Native | Decrease | *Orbaceae* |
| Genus 871 | Native | Decrease | *Flavobacteriaceae* |
| Genus 874 | Native | Decrease | *Enterobacteriaceae* |
| Genus 877 | Native | Decrease | *Burkholderiaceae* |
| Genus 883 | Native | Decrease | *Propionibacteriaceae* |
| Genus 904 | Native | Decrease | *Microbacteriaceae* |
| Genus 911 | Native | Decrease | *Staphylococcaceae* |
| Genus 927 | Native | Decrease | *Babeliales* |
| Genus 100 | Native | Increase | *Lysinibacillus* |
| Genus 149 | Native | Increase | *Enterobacteriaceae* |
| Genus 192 | Native | Increase | *Salmonella* |
| Genus 223 | Native | Increase | *Paraclostridium* |
| Genus 263 | Native | Increase | *Clostridium sensu stricto 1* |
| Genus 345 | Native | Increase | *Ignavibacteria* |
| Genus 448 | Native | Increase | *Clostridiales* |
| Genus 457 | Native | Increase | *Clostridioides* |
| Genus 526 | Native | Increase | *Clostridium sensu stricto 13* |
| Genus 669 | Native | Increase | *Tissierella* |
| Genus 998 | Native | Increase | *Terrisporobacter* |
| Genus 1 | Non-native | Decrease | *Blastocatellaceae* |
| Genus 2 | Non-native | Decrease | *Sphingomonadaceae* |
| Genus 3 | Non-native | Decrease | *Xanthomonadaceae* |
| Genus 4 | Non-native | Decrease | *Micrococcaceae* |
| Genus 8 | Non-native | Decrease | *Acidobacteria Subgroup 6* |
| Genus 11 | Non-native | Decrease | *Burkholderiaceae* |
| Genus 12 | Non-native | Decrease | *Nitrospira* |
| Genus 14 | Non-native | Decrease | *Betaproteobacteriales* |
| Genus 15 | Non-native | Decrease | *Betaproteobacteriales* |
| Genus 17 | Non-native | Decrease | *Gammaproteobacteria* |
| Genus 21 | Non-native | Decrease | *Acidobacteria Subgroup 6* |
| Genus 23 | Non-native | Decrease | *Bradyrhizobium* |
| Genus 24 | Non-native | Decrease | *Chitinophagaceae* |
| Genus 25 | Non-native | Decrease | *Burkholderiaceae* |
| Genus 26 | Non-native | Decrease | *Gemmatimonadaceae* |
| Genus 28 | Non-native | Decrease | *Nocardioides* |
| Genus 31 | Non-native | Decrease | *Saccharimonadales* |
| Genus 32 | Non-native | Decrease | *Nitrosomonadaceae* |
| Genus 33 | Non-native | Decrease | *Acidobacteria Subgroup 6* |
| Genus 36 | Non-native | Decrease | *Acidobacteria Subgroup 6* |
| Genus 37 | Non-native | Decrease | *Microscillaceae* |
| Genus 38 | Non-native | Decrease | *Microbacteriaceae* |
| Genus 39 | Non-native | Decrease | *Sphingomonas* |
| Genus 41 | Non-native | Decrease | *Planococcaceae* |
| Genus 42 | Non-native | Decrease | *Acidobacteria Subgroup 6* |
| Genus 43 | Non-native | Decrease | *Roseiflexaceae* |
| Genus 44 | Non-native | Decrease | *Chloroflexaceae* |
| Genus 49 | Non-native | Decrease | *Acidobacteria* |
| Genus 52 | Non-native | Decrease | *Bryobacter* |
| Genus 56 | Non-native | Decrease | *Microscillaceae* |
| Genus 65 | Non-native | Decrease | *Ilumatobacter* |
| Genus 68 | Non-native | Decrease | *Nitrosomonadaceae* |
| Genus 70 | Non-native | Decrease | *Betaproteobacteriales* |
| Genus 71 | Non-native | Decrease | *Myxococcales* |
| Genus 73 | Non-native | Decrease | *Nitrosomonadaceae* |
| Genus 75 | Non-native | Decrease | *Steroidobacter* |
| Genus 77 | Non-native | Decrease | *Rhizobiales* |
| Genus 78 | Non-native | Decrease | *Acidimicrobiia* |
| Genus 79 | Non-native | Decrease | *Anaerolineae* |
| Genus 81 | Non-native | Decrease | *Rokubacteriales* |
| Genus 82 | Non-native | Decrease | *Ensifer* |
| Genus 85 | Non-native | Decrease | *Microtrichales* |
| Genus 87 | Non-native | Decrease | *Candidatus Saccharibacteria* |
| Genus 96 | Non-native | Decrease | *Micrococcales* |
| Genus 103 | Non-native | Decrease | *Flavitalea* |
| Genus 107 | Non-native | Decrease | *Chloroflexi* |
| Genus 109 | Non-native | Decrease | *Lysobacter* |
| Genus 115 | Non-native | Decrease | *Comamonas* |
| Genus 119 | Non-native | Decrease | *Candidatus Udaeobacter* |
| Genus 128 | Non-native | Decrease | *Rhizobiales* |
| Genus 129 | Non-native | Decrease | *Haliangium* |
| Genus 130 | Non-native | Decrease | *Xanthobacteraceae* |
| Genus 131 | Non-native | Decrease | *Roseimicrobium* |
| Genus 134 | Non-native | Decrease | *Pseudolabrys* |
| Genus 141 | Non-native | Decrease | *Ramlibacter* |
| Genus 147 | Non-native | Decrease | *Paracoccus* |
| Genus 158 | Non-native | Decrease | *Acidobacteria Subgroup 17* |
| Genus 163 | Non-native | Decrease | *Gemmatimonas* |
| Genus 164 | Non-native | Decrease | *Actinomarinales* |
| Genus 168 | Non-native | Decrease | *Hevea brasiliensis* |
| Genus 170 | Non-native | Decrease | *Burkholderia-Caballeronia-Paraburkholderia* |
| Genus 174 | Non-native | Decrease | *Desulfarculaceae* |
| Genus 183 | Non-native | Decrease | *Hirschia* |
| Genus 191 | Non-native | Decrease | *Actinobacteria* |
| Genus 196 | Non-native | Decrease | *Bdellovibrio* |
| Genus 197 | Non-native | Decrease | *Caulobacter* |
| Genus 198 | Non-native | Decrease | *Pedobacter* |
| Genus 201 | Non-native | Decrease | *Alphaproteobacteria* |
| Genus 203 | Non-native | Decrease | *Saccharimonadales* |
| Genus 206 | Non-native | Decrease | *Rhodococcus* |
| Genus 209 | Non-native | Decrease | *Ferruginibacter* |
| Genus 210 | Non-native | Decrease | *Allorhizobium-Neorhizobium-Pararhizobium-Rhizobium* |
| Genus 212 | Non-native | Decrease | *Flavobacterium* |
| Genus 224 | Non-native | Decrease | *Nitrosomonadaceae* |
| Genus 226 | Non-native | Decrease | *Chloroplast* |
| Genus 233 | Non-native | Decrease | *Pseudofulvimonas* |
| Genus 244 | Non-native | Decrease | *Beijerinckiaceae* |
| Genus 246 | Non-native | Decrease | *Flavisolibacter* |
| Genus 253 | Non-native | Decrease | *Chitinophagaceae* |
| Genus 255 | Non-native | Decrease | *Gaiella* |
| Genus 256 | Non-native | Decrease | *Candidatus Solibacter* |
| Genus 285 | Non-native | Decrease | *Iamia* |
| Genus 286 | Non-native | Decrease | *Achromobacter* |
| Genus 293 | Non-native | Decrease | *Rhodoplanes* |
| Genus 311 | Non-native | Decrease | *Acidobacteriales* |
| Genus 316 | Non-native | Decrease | *Babeliales* |
| Genus 322 | Non-native | Decrease | *Brevundimonas* |
| Genus 323 | Non-native | Decrease | *Calothrix UAM 374* |
| Genus 329 | Non-native | Decrease | *Rhizobiales* |
| Genus 335 | Non-native | Decrease | *Chitinophaga* |
| Genus 340 | Non-native | Decrease | *Microbacterium* |
| Genus 352 | Non-native | Decrease | *Conexibacter* |
| Genus 355 | Non-native | Decrease | *Pajaroellobacter* |
| Genus 361 | Non-native | Decrease | *Coxiella* |
| Genus 377 | Non-native | Decrease | *Rhodobacteraceae* |
| Genus 394 | Non-native | Decrease | *Patescibacteria* |
| Genus 397 | Non-native | Decrease | *Frankiales* |
| Genus 415 | Non-native | Decrease | *Noviherbaspirillum* |
| Genus 436 | Non-native | Decrease | *Legionella* |
| Genus 437 | Non-native | Decrease | *Erysipelotrichaceae* |
| Genus 444 | Non-native | Decrease | *Sphingobacteriaceae* |
| Genus 445 | Non-native | Decrease | *Vermiphilaceae* |
| Genus 459 | Non-native | Decrease | *Oligoflexales* |
| Genus 460 | Non-native | Decrease | *Holophagae Subgroup 7* |
| Genus 461 | Non-native | Decrease | *Enterococcus* |
| Genus 462 | Non-native | Decrease | *Actinocorallia* |
| Genus 471 | Non-native | Decrease | *Oligoflexaceae* |
| Genus 474 | Non-native | Decrease | *Bacillales* |
| Genus 491 | Non-native | Decrease | *Truepera* |
| Genus 494 | Non-native | Decrease | *Bacillaceae* |
| Genus 523 | Non-native | Decrease | *Fibrobacteraceae* |
| Genus 525 | Non-native | Decrease | *Phenylobacterium* |
| Genus 530 | Non-native | Decrease | *Acidobacteriales* |
| Genus 536 | Non-native | Decrease | *Mucilaginibacter* |
| Genus 550 | Non-native | Decrease | *Sandaracinus* |
| Genus 555 | Non-native | Decrease | *Edaphobacter* |
| Genus 563 | Non-native | Decrease | *Patulibacter* |
| Genus 564 | Non-native | Decrease | *Vulgatibacter* |
| Genus 565 | Non-native | Decrease | *Acetobacteraceae* |
| Genus 572 | Non-native | Decrease | *Acidobacteriia Subgroup 2* |
| Genus 579 | Non-native | Decrease | *Myroides* |
| Genus 582 | Non-native | Decrease | *Rhodanobacter* |
| Genus 591 | Non-native | Decrease | *Staphylococcus* |
| Genus 598 | Non-native | Decrease | *Acidithiobacillaceae* |
| Genus 619 | Non-native | Decrease | *Granulicella* |
| Genus 632 | Non-native | Decrease | *Cohnella* |
| Genus 634 | Non-native | Decrease | *Enhydrobacter* |
| Genus 645 | Non-native | Decrease | *Corynebacterium 1* |
| Genus 653 | Non-native | Decrease | *Moraxellaceae* |
| Genus 656 | Non-native | Decrease | *Serratia* |
| Genus 664 | Non-native | Decrease | *Mitochondria* |
| Genus 666 | Non-native | Decrease | *Cupriavidus* |
| Genus 668 | Non-native | Decrease | *Wohlfahrtiimonas* |
| Genus 680 | Non-native | Decrease | *Alcaligenes* |
| Genus 682 | Non-native | Decrease | *Providencia* |
| Genus 685 | Non-native | Decrease | *Weeksellaceae* |
| Genus 692 | Non-native | Decrease | *Spirosomaceae* |
| Genus 695 | Non-native | Decrease | *Ignavibacteria* |
| Genus 702 | Non-native | Decrease | *Telmatospirillum* |
| Genus 716 | Non-native | Decrease | *Streptococcus* |
| Genus 720 | Non-native | Decrease | *Moheibacter* |
| Genus 764 | Non-native | Decrease | *Solanum melongena* |
| Genus 766 | Non-native | Decrease | *Xanthomonadales* |
| Genus 858 | Non-native | Decrease | *Halomonadaceae* |
| Genus 860 | Non-native | Decrease | *Proteiniphilum* |
| Genus 871 | Non-native | Decrease | *Flavobacteriaceae* |
| Genus 891 | Non-native | Decrease | *Actinomyces* |
| Genus 897 | Non-native | Decrease | *Desulfotomaculum* |
| Genus 904 | Non-native | Decrease | *Leucobacter* |
| Genus 9 | Non-native | Increase | *Acinetobacter* |
| Genus 30 | Non-native | Increase | *Pseudomonas* |
| Genus 66 | Non-native | Increase | *Bacillus* |
| Genus 100 | Non-native | Increase | *Lysinibacillus* |
| Genus 263 | Non-native | Increase | *Clostridium sensu stricto 1* |
| Genus 448 | Non-native | Increase | *Clostridiales* |
| Genus 457 | Non-native | Increase | *Clostridioides* |
| Genus 526 | Non-native | Increase | *Clostridium sensu stricto 13* |
| Genus 833 | Non-native | Increase | *Sporacetigenium* |

**Table S9** The specific bacterial functional groups associated with earthworm decomposition at different sampling times.

|  | Native soil | | | | | | | | | | | | | | | | | | | | Non-native soil | | | | | | | | | | | | | | | | | | | |
| --- | --- | --- | --- | --- | --- | --- | --- | --- | --- | --- | --- | --- | --- | --- | --- | --- | --- | --- | --- | --- | --- | --- | --- | --- | --- | --- | --- | --- | --- | --- | --- | --- | --- | --- | --- | --- | --- | --- | --- | --- |
|  | 1 day | | | | | 3 day | | | | | 5 day | | | | | 8 day | | | | | 1 day | | | | | 3 day | | | | | 5 day | | | | | 8 day | | | | |
|  | R1 | R2 | R3 | R4 | R5 | R1 | R2 | R3 | R4 | R5 | R1 | R2 | R3 | R4 | R5 | R1 | R2 | R3 | R4 | R5 | R1 | R2 | R3 | R4 | R5 | R1 | R2 | R3 | R4 | R5 | R1 | R2 | R3 | R4 | R5 | R1 | R2 | R3 | R4 | R5 |
| F1 | - | - | - | - |  |  |  |  |  |  | - | - | - | - | - |  |  |  |  |  |  |  |  |  |  | - | - | - |  |  |  |  |  |  |  |  |  |  |  |  |
| F2 | - | - | - | - |  |  |  |  |  |  | - | - | - | - | - | - |  |  |  |  |  |  |  |  |  |  |  |  |  |  |  |  |  |  |  | - |  |  |  | - |
| F3 |  |  |  |  |  |  |  |  |  |  | - | - | - | - | - |  |  |  |  |  | - | - | - | - | - |  |  |  | - | - | - | - |  |  |  | - | - |  | - | - |
| F4 |  |  |  |  |  |  |  |  |  |  | - | - | - | - | - |  |  |  |  |  |  |  | - |  | - |  |  |  |  |  | - | - |  |  |  | - | - |  | - | - |
| F5 |  |  |  |  |  |  |  |  |  |  | - | - | - | - | - |  |  |  |  |  |  |  | - |  | - |  |  |  |  |  | - | - |  |  |  | - | - |  | - | - |
| F6 | - | - |  |  |  | - | - |  |  |  | + | + | + | + | + | + |  |  |  |  |  |  |  |  |  | - | - | - |  |  |  |  | - |  |  |  | + | + | + | + |
| F7 |  |  |  |  |  |  |  |  |  |  |  |  |  |  |  |  |  |  |  |  |  | + | + | + | + | + | + | + | + | + |  |  |  | + | + |  |  |  |  |  |
| F8 | + | + |  | + |  | + | + |  | + |  | + | + |  | + |  | + | + |  | + |  |  |  |  |  | - |  |  |  |  |  |  |  |  |  |  |  |  |  |  |  |
| F9 |  |  |  |  |  |  |  | + |  | + |  |  |  |  |  |  |  |  |  |  |  |  |  |  |  |  |  |  |  |  |  |  |  |  |  |  |  |  |  |  |
| F10 |  |  |  |  |  |  | + | + | + | + |  |  |  |  |  |  |  |  |  |  |  |  | + |  | + | + | + | + | + | + |  |  |  |  |  |  |  |  |  |  |
| F11 | + | + |  | + |  | + | + |  | + |  |  |  |  | + |  |  |  |  |  |  |  |  |  |  |  |  |  |  |  |  |  |  |  |  |  |  |  |  |  |  |
| F12 | + | + |  | + |  | + | + |  | + |  |  |  |  | + |  |  |  |  |  |  |  |  |  |  |  |  |  |  |  |  |  |  |  |  |  |  |  |  |  |  |
| F13 | + | + | + | + | + |  |  |  |  |  |  |  |  |  |  |  |  |  |  |  |  |  |  |  |  | + | + | + | + | + | + | + |  | + | + |  |  |  |  |  |
| F14 | + | + | + | + | + |  |  |  |  |  |  |  |  |  |  |  |  |  |  |  |  |  |  |  |  | + | + | + | + | + | + | + |  | + | + |  |  |  |  |  |
| F15 | + | + | + | + | + |  |  |  |  |  | + | + |  | + |  |  |  |  |  |  |  |  |  |  |  |  |  |  |  |  |  |  |  |  |  |  |  |  |  |  |
| F16 | + | + |  | + |  |  |  |  | + |  |  |  |  |  |  |  |  |  |  |  |  |  |  |  |  |  | + | + | + | + |  |  |  | + | + |  |  |  |  |  |
| F17 | + | + |  | + |  |  |  |  | + |  |  |  |  |  |  |  |  |  |  |  |  |  |  |  |  |  | + | + | + | + |  |  |  | + | + |  |  |  |  |  |
| F18 | + | + |  | + |  |  |  |  | + |  |  |  |  |  |  |  |  |  |  |  |  |  |  |  |  |  | + | + | + | + |  |  |  | + | + |  |  |  |  |  |
| F19 | + | + |  | + |  |  |  |  | + |  |  |  |  |  |  |  |  |  |  |  |  |  |  |  |  |  | + | + | + | + |  |  |  | + | + |  |  |  |  |  |
| F20 | + | + |  | + |  |  |  |  | + |  |  |  |  |  |  |  |  |  |  |  |  |  |  |  |  |  | + | + | + | + |  |  |  | + | + |  |  |  |  |  |
| F21 | + | + |  | + |  |  |  |  | + |  |  |  |  |  |  |  |  |  |  |  |  |  |  |  |  |  | + | + | + | + |  |  |  | + | + |  |  |  |  |  |
| F22 |  |  |  |  |  |  |  |  |  | - |  |  |  |  |  |  |  |  |  |  | - | - | - | - | - | - | - | - | - | - |  |  |  | - |  |  |  |  |  |  |
| F23 |  |  |  |  |  |  |  |  |  |  |  |  |  |  |  |  |  |  |  |  |  |  |  |  |  |  |  |  |  |  |  |  |  |  |  |  | - |  | - | - |
| F24 |  |  |  |  |  |  |  |  |  |  |  |  |  |  |  |  |  |  |  |  |  |  |  |  |  |  |  |  |  |  | - |  |  | - |  |  |  |  |  |  |
| F25 |  |  |  |  |  |  |  | - | - | - |  |  |  |  |  |  |  |  |  |  |  |  |  |  |  | + | + |  |  |  | + | + |  |  |  |  |  |  |  |  |
| F26 |  |  |  |  |  |  |  |  |  |  |  |  |  |  |  |  |  |  |  |  | - | - | - | - | - | - | - |  | - |  |  |  |  |  |  |  |  |  |  |  |
| F27 |  |  |  |  |  | + | + |  | + |  | + |  |  |  |  |  |  |  |  |  | - | - | - | - | - | - | - |  | - | - |  |  |  |  |  |  |  |  |  |  |
| F28 | - | - | - | - | - | - | - | - | - | - | - | - |  | - |  |  |  |  |  |  | + | + | + |  |  | + | + |  |  |  | + | + |  |  |  |  |  |  |  |  |
| F29 | - | - | - | - | - | - | - | - | - | - | - | - |  | - |  |  |  |  |  |  | + | + | + |  |  | + | + |  |  |  | + | + |  |  |  |  |  |  |  |  |
| F30 | - | - | - | - | - | - | - | - | - | - | - | - |  | - |  |  |  |  |  |  | + | + | + |  |  | + | + |  |  |  | + | + |  |  |  |  |  |  |  |  |
| F31 |  |  |  |  |  | - | - |  | - |  |  |  |  |  |  |  |  |  |  |  | - | - | - | - | - | - | - | - | - | - |  |  |  |  |  |  |  |  |  |  |
| F32 |  |  |  |  |  |  |  |  |  |  | + | + |  |  |  |  |  |  |  |  |  |  |  |  |  |  |  |  |  |  |  |  |  |  |  |  |  |  |  |  |
| F33 |  |  |  |  |  |  |  |  |  |  | + | + |  |  |  |  |  |  |  |  |  |  |  |  |  |  |  |  |  |  |  |  |  |  |  |  |  |  |  |  |
| F34 | + | + | + | + |  | + | + | + | + | + |  |  |  |  |  |  |  |  |  |  |  |  | - |  | - |  |  |  |  |  |  |  |  | - | - |  |  |  |  |  |
| F35 |  |  |  |  |  |  |  |  |  |  |  |  |  |  |  | + | + |  | + |  | - | - | - | - | - | - | - |  | - | - |  |  |  | - | - |  |  |  |  |  |
| F36 | + | + |  | + |  | + | + | + | + |  | + | + |  | + |  |  |  |  |  |  |  | - | - | - | - |  |  |  | - |  |  |  |  | - | - |  |  |  | - |  |
| F37 | + | + |  | + |  | + | + | + | + |  | + | + |  | + |  |  |  |  |  |  |  | - | - | - | - |  |  |  | - |  |  |  |  | - | - |  |  |  | - |  |
| F38 |  |  |  |  |  |  |  |  |  |  |  |  |  |  |  |  |  |  |  |  | - | - | - | - | - |  |  |  |  |  |  |  |  |  |  |  |  |  |  |  |
| F39 |  |  |  |  |  |  |  |  |  |  |  |  |  |  |  |  |  |  |  |  |  |  |  |  |  |  |  |  |  |  |  |  |  |  |  |  |  |  |  |  |
| F40 |  |  |  |  |  |  |  |  |  |  |  |  |  |  |  |  |  |  |  |  |  |  |  |  |  |  |  |  |  |  |  |  |  |  |  |  |  |  |  |  |
| F41 | - | - | - | - | - | - | - |  | - |  |  |  |  |  |  |  |  |  |  |  |  |  | + | + | + |  |  | + |  | + |  |  |  |  |  |  |  |  |  |  |
| F42 | - | - | - | - | - | - | - |  | - |  |  |  |  |  |  |  |  |  |  |  |  |  | + | + | + |  |  | + |  | + |  |  |  |  |  |  |  |  |  |  |

R1: Region 1; R2: Region 2; R3: Region 3; R4: Region 4; R5: Region 5; F1: chemoheterotrophy; F2: aerobic_chemoheterotrophy; F3: aromatic_compound_degradation; F4: animal_parasites_or_symbionts; F5: human_pathogens_all; F6: fermentation; F7: chloroplasts; F8: nitrate_reduction; F9: manganese_oxidation; F10: intracellular_parasites; F11: nitrate_respiration; F12: nitrogen_respiration; F13: methanol_oxidation; F14: methylotrophy; F15: predatory_or_exoparasitic; F16: nitrate_denitrification; F17: nitrite_denitrification; F18: nitrous_oxide_denitrification; F19: denitrification; F20: dark_hydrogen_oxidation; F21: nitrite_respiration; F22: respiration_of_sulfur_compounds; F23: cellulolysis; F24: chitinolysis; F25: phototrophy; F26: ureolysis; F27: nitrogen_fixation; F28:cyanobacteria; F29: oxygenic_photoautotrophy; F30: photoautotrophy; F31: sulfur_respiration; F32: aerobic_nitrite_oxidation; F33: nitrification; F34: hydrocarbon_degradation; F35: sulfate_respiration; F36: aromatic_hydrocarbon_degradation; F37: aliphatic_non_methane_hydrocarbon_degradation; F38: photoheterotrophy; F39: dark_sulfide_oxidation; F40: dark_oxidation_of_sulfur_compounds; F41: human_gut; F42: mammal_gut. +: positive correlation; -: negative correlation; blank spaces indicate no correlation.
